# Dynamics of RNA localization to nuclear speckles are connected to splicing efficiency

**DOI:** 10.1126/sciadv.adp7727

**Published:** 2024-10-16

**Authors:** Jinjun Wu, Yu Xiao, Yunzheng Liu, Li Wen, Chuanyang Jin, Shun Liu, Sneha Paul, Chuan He, Oded Regev, Jingyi Fei

**Affiliations:** ^1^ Department of Biochemistry and Molecular Biology, The University of Chicago, Chicago, IL 60637, USA.; ^2^ Department of Chemistry, The University of Chicago, Chicago, IL 60637, USA.; ^3^ Institute for Biophysical Dynamics, The University of Chicago, Chicago, IL 60637, USA.; ^4^ Howard Hughes Medical Institute, The University of Chicago, 929 East 57th Street, Chicago, IL 60637, USA.; ^5^ Department of Physics, The University of Chicago, Chicago, IL 60637, USA.; ^6^ Courant Institute of Mathematical Sciences, New York University, New York, NY 10012, USA.

## Abstract

Nuclear speckles are nuclear membraneless organelles in higher eukaryotic cells playing a vital role in gene expression. Using an in situ reverse transcription–based sequencing method, we study nuclear speckle–associated human transcripts. Our data indicate the existence of three gene groups whose transcripts demonstrate different speckle localization properties: stably enriched in nuclear speckles, transiently enriched in speckles at the pre–messenger RNA stage, and not enriched. We find that stably enriched transcripts contain inefficiently excised introns and that disruption of nuclear speckles specifically affects splicing of speckle-enriched transcripts. We further reveal RNA sequence features contributing to transcript speckle localization, indicating a tight interplay between transcript speckle enrichment, genome organization, and splicing efficiency. Collectively, our data highlight a role of nuclear speckles in both co- and posttranscriptional splicing regulation. Last, we show that genes with stably enriched transcripts are over-represented among genes with heat shock–up-regulated intron retention, hinting at a connection between speckle localization and cellular stress response.

## Teaser

ARTR-seq reveals properties of transcripts localizing to nuclear speckles and connections to RNA splicing.

## INTRODUCTION

Membraneless organelles are prevalent in eukaryotic cells and are broadly involved in processing and assembling ribonucleoprotein complexes, gene expression, signal transduction, and stress responses (*1*). Nuclear speckles are a type of membraneless organelle in the nucleus of higher eukaryotic cells. A typical cell contains tens of nuclear speckles, ranging in size from a few hundred nanometers to a few micrometers. Nuclear speckles have a rich proteome consisting of more than a hundred protein species, many of which are spliceosomal components or splicing regulators (*2*, *3*). Their core region is defined by the two scaffold proteins SON (SON DNA and RNA binding protein) and SRRM2 (serine/arginine repetitive matrix 2) (*4*). Nuclear speckles also have a rich transcriptome, including polyadenylated [poly (A)^+^] RNAs and certain long noncoding RNAs (lncRNAs), as was recently systematically mapped through APEX-seq, an RNA sequencing method based on proximity labeling using peroxidase enzyme APEX2 (*5*). Changes in nuclear speckle composition or morphology are frequently associated with cancers, neuronal disorders, and infection (*2*, *6*–*8*). However, the fundamental roles of nuclear speckles in gene expression remain elusive, making it challenging to mechanistically connect nuclear speckles to pathogenesis of these diseases.

While nuclear speckles were historically considered as storage sites for splicing factors, new evidence now portrays nuclear speckles as active hubs promoting gene expression (*2*, *3*, *9*). A positive correlation has been observed between expression level and the speckle proximity of gene foci, both in fluorescence imaging using in situ hybridization (*10*, *11*) and in more recent genome-wide sequencing through proximity labeling (*12*–*14*). It was suggested that around 50% of transcriptionally active genes are associated with nuclear speckles (*13*). However, it is unclear why certain genes are associated with speckles while others are not. Gene foci have also been observed to localize close to nuclear speckles in a regulated fashion. For example, α-globin and β-globin genes are localized to nuclear speckles when actively transcribed during erythroid differentiation (*15*), and *HSPA1A* transgenes were observed to move toward nuclear speckles upon heat shock (*16*). Moreover, as demonstrated with p53, transcription factors can drive localization of a subset of their target genes to nuclear speckles, enhancing their RNA expression levels (*17*).

Being a compartment enriched in splicing factors (*2*, *3*), nuclear speckles are tightly linked to splicing. Detection within nuclear speckles of phosphorylated SF3b, considered as a marker for active spliceosomes (*18*–*20*), suggests active splicing taking place in speckles (*21*). The enhanced enrichment of poly (A)^+^ RNAs in speckles upon splicing inhibition is indicative of speckles as a compartment to accommodate posttranscriptionally accumulated transcripts (*21*, *22*). In line with this observation, the recent APEX-seq mapping of nuclear speckle–localized transcriptome revealed an enrichment of retained introns in nuclear speckles (*5*). These results suggest that nuclear speckles serve as a posttranscriptional quality control compartment for incompletely spliced transcripts. In addition, nuclear speckles were demonstrated to promote cotranscriptional splicing through increased binding of spliceosomes to pre-mRNAs from speckle-proximal genes (*11*, *23*). In addition to promoting constitutive splicing, nuclear speckles were also demonstrated to facilitate splicing of stress-related genes under ribotoxic stress in a regulated fashion (*24*), as well as affecting alternative splicing (*25*). However, within these proposed functions of nuclear speckle in splicing, fundamental questions are not addressed: (i) Do all genes require speckles for co- or posttranscriptional splicing? (ii) If not, do transcripts using speckles for co- or posttranscriptional splicing differ in any way? Addressing these questions will provide us with a clearer framework of how nuclear speckles coordinate co- and posttranscriptional splicing.

In this work, we use ARTR-seq (reverse transcription–based RNA binding protein binding sites sequencing) (*26*) to comprehensively quantify the speckle transcriptome. We identify three gene groups whose transcripts demonstrate different speckle localization properties: Transcripts from group A genes are stably enriched in nuclear speckles; transcripts from group B genes are transiently enriched in speckles at the pre-mRNA stage; and transcripts from group C genes are not enriched in speckles. Through a biochemical assay, we demonstrate a functional role of nuclear speckles in promoting splicing of speckle-associated transcripts from group A and B genes. We further reveal RNA sequence cis-elements that contribute to transcript speckle localization, suggesting a tight interplay between gene position, sequence features, and transcript speckle enrichment. Last, using heat shock as an example, we suggest that nuclear speckles may accommodate transcripts undergoing up-regulated intron retention during cellular stress.

## RESULTS

### ARTR-seq identifies nuclear speckle transcriptome

**Fig. 1.**
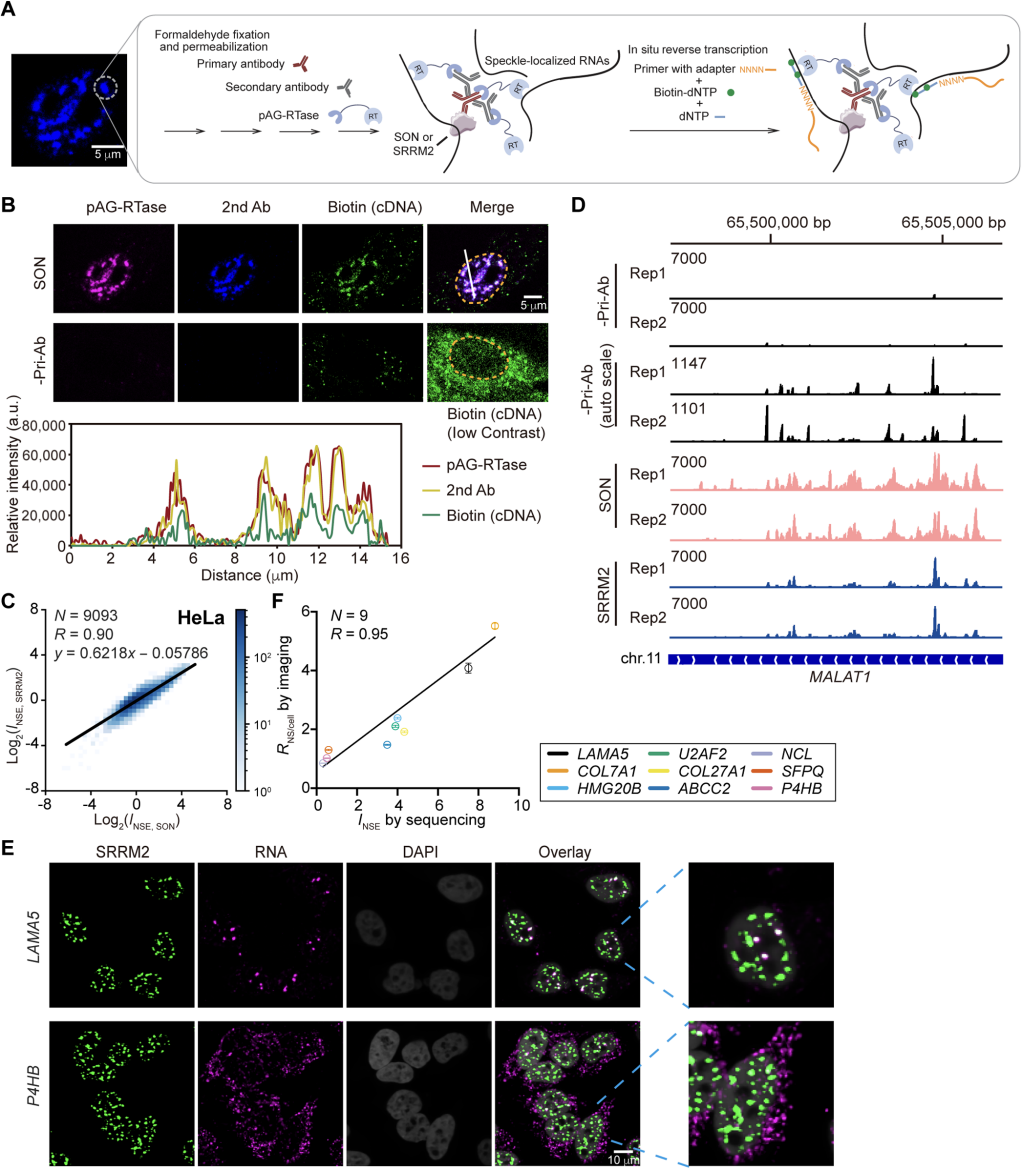
Characterization of nuclear speckle–enriched transcriptome using ARTR-seq. (**A**) Scheme of ARTR-seq: Scaffold protein is immunostained by primary and secondary antibodies (Abs) sequentially. pAG-RTase then binds to the antibody to initiate reverse transcription in situ. The generated biotinylated cDNAs are collected and prepared for sequencing. (**B**) Representative image showing colocalization of Alexa Fluor 647 (AF647)–labeled pAG-RTase (magenta), Alexa Fluor 568 (AF568)–labeled secondary antibody against anti-SON primary antibody (blue), and biotinylated cDNA detected by Alexa Fluor 488 (AF488)–labeled antibody against biotin (green). The biotinylated cDNA signal in the image without the use of primary antibody (-Pri-Ab) is also shown at a fivefold lower contrast. Dashed line marks the nucleus boundary. (**C**) Two-dimensional (2D) histogram showing the correlation between *I*_NSE_ determined through targeting SON and SRRM2 (in log_2_ scale) in HeLa cells. Genes with *lfcSE* < 1 from DESeq analysis of ARTR-seq are included. Linear function (*y* = *ax* + *b*) is used to fit *I*_NSE,SRRM2_ to *I*_NSE,SON_. (**D**) Genome tracks showing ARTR-seq reads generated from targeting SON or SRRM2 and from samples without primary antibody (-Pri-Ab), mapped to *MALAT1* gene. Additional genome track for -Pri-Ab is shown using autoscale for the number of reads. (**E**) RNA FISH images showing *LAMA5* and *P4HB* transcript. RNA FISH probes were labeled with AF647 (magenta). Nuclear speckles were immunostained with AF488 (green). Nuclei were stained with 4′,6-diamidino-2-phenylindole (DAPI) (gray). The zoomed-in image shows one nucleus in each case. (**F**) Correlation between speckle partition coefficient (*R*_NS/cell_) measured by RNA FISH imaging and *I*_NSE_ values determined by ARTR-seq. *R*_NS/cell_ was calculated as the ratio of fluorescence signal inside nuclear speckles to that in the entire cell, which should theoretically correspond to *I*_NSE_. In (C) and (F), “*N*” reports the number of genes included in the analysis, and “*R*” reports Pearson’s correlation coefficient.

To map the nuclear speckle–enriched transcriptome, we adopted our recently developed method ARTR-seq (*26*). This method uses a recombinant enzyme consisting of Protein A/G fused to a reverse transcriptase (pAG-RTase) (Fig. 1A). Protein A/G combines the immunoglobulin G (IgG) binding domains of protein A and protein G and can thus bind to most IgG subclasses including polyclonal or monoclonal IgG antibodies. Because the physical distance between the RTase and the targeted protein is estimated to be at most ~45 nm, considering the physical dimension of antibodies (~14 nm), pAG (~3 nm), RTase (~4 nm), and the 30–amino acid linker in between (~10 nm) (*27*), we reasoned that the method is well suited for identification of the transcriptome within membraneless organelles. Following cell fixation and permeabilization, a nuclear speckle scaffold protein (either SON or SRRM2) was first labeled with primary and secondary antibodies and then pAG-RTase. Reverse transcription was initiated by exogenous addition of biotin deoxynucleotide triphosphates (dNTP), random 10-mer primers with additional adapter, and other reaction components, followed by cell lysis, ribonuclease (RNase) treatment, and affinity enrichment of biotinylated cDNAs using streptavidin beads. The biotinylated cDNAs were ligated with an adapter and prepared for library amplification and sequencing. The fluorophore-labeled pAG-RTase showed good colocalization with antibodies against SON and biotin-labeled cDNA (Fig. 1B), with all signals exhibiting specific nuclear speckle localization. This colocalization analysis confirms that pAG-RTase can effectively perform reverse transcription in situ with the desired spatial localization. We denote the number of reads mapped to each gene (whether in exons or in introns) as *N*_SON_ when SON is targeted and as *N*_SRRM2_ when SRRM2 is targeted.

To calculate the nuclear speckle enrichment index (*I*_NSE_) for each gene, we performed ARTR-seq without a primary antibody (reads denoted by *N*_-priAB_). Under these conditions, the secondary antibody, pAG-RTase, and generated biotinylated cDNA exhibited weak diffusive signals throughout the cell (Fig. 1B). Because the amount of biotinylated cDNA generated upon nonspecific binding of secondary antibody and pAG-RTase is expected to be dependent on the surrounding transcript concentration, we interpret *N*_-priAB_ as reflecting the average cellular transcript concentration. We then calculated *I*_NSE_ for each gene using differential analysis between *N*_SON_ (or *N*_SRRM2_) and *N*_-priAB_ (Fig. 1C). Theoretically, *I*_NSE_ should reflect the ratio between the RNA concentration inside nuclear speckles and that in the cell. Like any sequencing-based method, ARTR-seq is likely to have some sequencing biases. However, we noticed similar read coverage patterns using both marker proteins and without antibody (Fig. 1D), suggesting that any sequence biases are largely eliminated in the differential analysis, with minimal impact on *I*_NSE_.

To demonstrate the robustness of our method to the choice of marker protein, we compared *I*_NSE_ values obtained from SON (*I*_NSE,SON_) to those obtained from SRRM2 (*I*_NSE,SRRM2_). We found that in both HeLa and HepG2 cells, *I*_NSE,SON_ and *I*_NSE,SRRM2_ show a high degree of correlation (Pearson’s correlation coefficient, 0.87 to 0.90; Fig. 1C and fig. S1A). Also, the well-known speckle-localized lncRNA *MALAT1* (*28*) was consistently identified with a high *I*_NSE_ with both marker proteins. This robustness of ARTR-seq to the choice of marker protein demonstrates that it can capture RNA in a defined three-dimensional proximity, largely representing speckle-localized RNAs.

While both marker proteins provided reproducible results, we did notice that *I*_NSE,SON_ spans a broader range of values compared to *I*_NSE,SRRM2_, which is reflected by a slope smaller than 1 when linearly fitting *I*_NSE,SRRM2_ to *I*_NSE,SON_ (Fig. 1C and fig. S1A). In addition, replicates of *I*_NSE,SON_ showed less variation compared to those of *I*_NSE,SRRM2_ (fig. S1B). Together, these comparisons suggest that measuring speckle enrichment by targeting SON generated stronger signal with less noise. Therefore, we mainly use *I*_NSE,SON_ for the rest of our analyses, with key results also reproduced using *I*_NSE,SRRM2_.

To validate the sequencing data, we performed fluorescence in situ hybridization (FISH) imaging on several RNA transcripts exhibiting a range of *I*_NSE_ values (Fig. 1E and fig. S1C). We used FISH probes targeting exonic regions to capture both spliced and unspliced transcripts of each selected gene. We calculated the ratio of fluorescence signal inside nuclear speckles to that in the entire cell, which should theoretically correspond to *I*_NSE_. The two quantities demonstrated a strong quantitative correlation (Pearson’s correlation coefficient, 0.95; Fig. 1F).

Last, we compared our ARTR-seq results with the previously reported APEX-seq results (fig. S2) (*5*). *I*_NSE,SON_ values demonstrate a positive correlation with the “Index 1” calculated from APEX-seq (Pearson’s correlation coefficient, 0.36 to 0.38; fig. S2). This index provides ordinal (rank) information on speckle enrichment. However, quantitative enrichment information is not easily derivable from the APEX-seq result. This is due to the exogenous APEX2 fusion protein potentially perturbing gene expression, which necessitates additional controls to confidently identify enriched genes. By avoiding the use of exogenous fusion proteins, ARTR-seq can directly provide enrichment quantification. In addition, the speckle marker proteins used in the APEX-seq study (SRSF1, SRSF7, and RNPS1) are not as highly enriched in speckles as the scaffold protein SON and SRRM2 (*29*, *30*). In summary, ARTR-seq provides robust and quantitative information on the nuclear speckle transcriptome.

### Unexcised introns are enriched in nuclear speckles

**Fig. 2.**
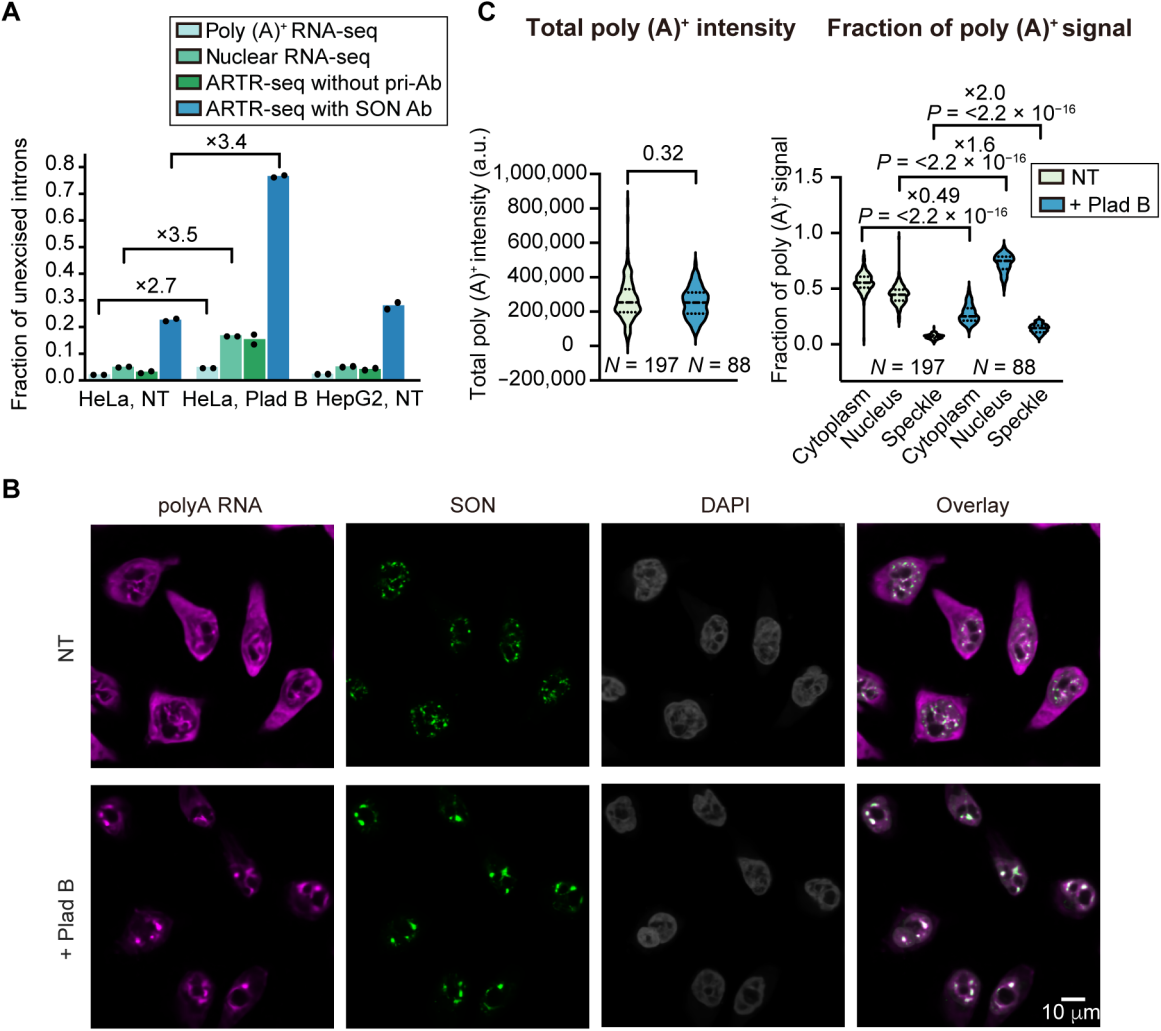
Plad B treatment increases the fraction of unspliced transcripts and speckle localization of poly (A)^+^ RNAs. (**A**) Fraction of unexcised introns [*EI*/(*EI* + *EE*)] calculated from the number of reads mapped to exon-intron boundary (*EI*) and to exon-exon junction (*EE*) in different RNA-seq samples. Each bar reports mean of two RNA-seq replicates. Fold change of fraction of unexcised introns upon Plad B treatment is indicated above each pair. (**B**) RNA FISH images of poly (A)^+^ RNAs using AF647-labeled polyT DNA probes (magenta) in the NT and Plad B treatment conditions. Nuclear speckles were immunostained with AF488 (green). Nuclei were stained with DAPI (gray). (**C**) Violin plots showing the total poly (A)^+^ intensity (left) and fraction of poly (A)^+^ signal in the cytoplasm, nucleus, and nuclear speckle (right). Fold change of poly (A)^+^ RNA fraction upon Plad B treatment is indicated above each pair. “*N*” reports number of cells included in the analysis, and *P* values are calculated with unpaired *t* tests in (C).

We next compared the total number of reads mapped to an exon-intron boundary (*EI*, averaged over both splice sites) to the total number of reads mapped to an exon-exon junction (*EE*). Combining these two values provides a global estimate of the fraction of unexcised introns [calculated as *EI*/(*EI* + *EE*)]. Under normal conditions [no treatment (NT)], we observed a similarly low fraction of unexcised introns in poly (A)^+^ RNA sequencing (RNA-seq), in ribosomal RNA (rRNA)–depleted nuclear RNA-seq, and in ARTR-seq without antibody, whereas the fraction of unexcised introns in ARTR-seq with SON antibody is about seven- to eightfold higher (Fig. 2A). This comparison suggests that in general, speckle-localized transcripts contain more unexcised introns compared to nucleus-localized transcripts.

### Splicing inhibition increases the amount of unspliced transcripts in nuclear speckles

To further establish the connection between splicing and speckle localization, we inhibited splicing by treating HeLa cells with 100 nM pladienolide B (Plad B) for 4 hours. Consistent with previous results (*31*), we observed an increase in speckle size (fig. S3, A and B), accompanied by a decrease in the number of speckles per cell (fig. S3C), indicating speckle fusion upon Plad B treatment. In addition, while total SON and SRRM2 abundance in the nucleus and nuclear speckle (as measured by total fluorescence intensity) is unaffected by Plad B treatment (fig. S3D), their concentrations in the nuclear speckle (as measured by average fluorescence intensity) (fig. S3E) and enrichment in speckle relative to nucleus (fig. S3F) increase consistently.

Poly (A)^+^ RNA-seq, nuclear RNA-seq, and ARTR-seq revealed a 2.7- to 3.5-fold increase in the fraction of unexcised introns upon Plad B treatment (Fig. 2A), pointing to a global increase in the fraction of unspliced transcripts. The similar fold change across the three RNA-seq libraries suggests that the speckle localization propensity, or speckle enrichment, of spliced transcripts (and similarly, also of unspliced transcripts) is globally unaffected by Plad B treatment despite a global shift toward unspliced transcripts.

We also observed that while the total poly (A)^+^ RNA signal (which captures both spliced and unspliced transcripts) does not change upon Plad B treatment, the fraction of nucleus-localized and nuclear speckle–localized poly (A)^+^ RNA signals increase disproportionately (Fig. 2, B and C), consistent with earlier reports (*22*). Because unspliced transcripts overall have higher speckle enrichment than spliced transcripts (Fig. 2A), we interpret the increase in speckle-localized poly (A)^+^ RNA fraction to be caused by the global increase of unspliced transcripts upon splicing inhibition. In other words, while the speckle localization propensity of unspliced or spliced transcripts remains unchanged, a global shift toward unspliced transcripts leads to the presence of more transcripts in speckles.

### Transcripts demonstrate diverse dynamics in speckle localization

To obtain a more detailed understanding of transcript speckle association, we introduced two further refinements to the *I*_NSE_ values. First, motivated by the Plad B–dependent changes in poly (A)^+^ RNA signal and in the fraction of unspliced transcripts observed above, we further calculated *I*_NSE_ values using either exon reads only (denoted as *I*_NSE(exon)_) or intron reads only (denoted as *I*_NSE(intron)_). Thus, *I*_NSE(exon)_ reflects speckle enrichment of “total transcripts” (both spliced and unspliced). In contrast, *I*_NSE(intron)_ reflects speckle enrichment of unspliced transcripts. We note that while some intronic reads might originate from excised intron lariats (or lariat intermediates), our data suggest that these reads are a minority and should not substantially affect our analyses (fig. S4); this is consistent with the rapid degradation of lariats (*32*).

Our second refinement is intended to capture transcripts that are transiently associated with nuclear speckles. It is suggested that most nascent transcripts are cotranscriptionally spliced (*33*, *34*). Because of the coupling between splicing and nuclear export (*35*, *36*), these rapidly spliced transcripts are subsequently exported. Any association of these transcripts with speckles during cotranscriptional splicing is therefore expected to be transient and will not be frequently captured by ARTR-seq. We therefore reasoned that inhibiting splicing with Plad B may allow us to extend the speckle localization time of these transcripts and better capture them in ARTR-seq. Moreover, our data below suggest that transcripts that do not localize to speckles under NT condition generally remain unassociated with speckles after Plad B treatment.

**Fig. 3.**
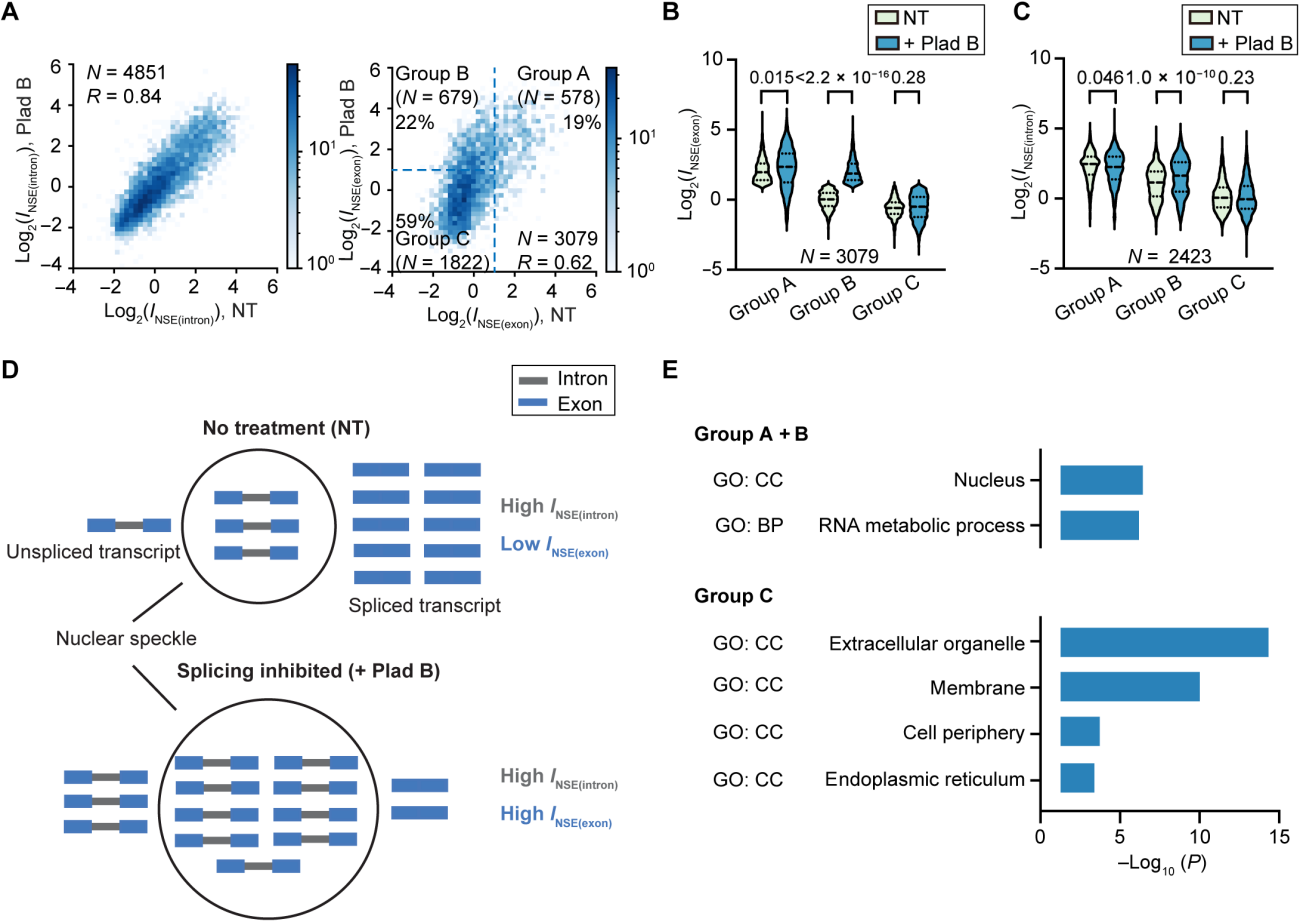
Transcripts exhibit varying nuclear speckle localization propensities and dynamics. (**A**) 2D histogram showing the correlation of *I*_NSE(intron)_ (left) or *I*_NSE(exon)_ (right) (in log_2_ scale) between NT and Plad B treatment conditions in HeLa cells. Group A, B, and C genes are depicted in the histogram of *I*_NSE(exon)_. (**B** and **C**) Violin plot comparing *I*_NSE(exon)_ (B) or *I*_NSE(intron)_ (C) values among group A, B, and C genes. (**D**) A model explaining the changes in *I*_NSE(intron)_ and *I*_NSE(exon)_ of group B transcripts upon Plad B treatment. Group B genes have a high speckle (black circle)–localized fraction of their pre-mRNAs (3 of 4 = 75% in this example), leading to a high *I*_NSE(intron)_. However, spliced transcripts are not enriched in speckles, leading to a low speckle-localized fraction of total transcripts (3 of 14 = 21.4%) and a low *I*_NSE(exon)_. Splicing inhibition does not change the fraction of unspliced transcripts in speckles (still 75%), explaining largely unchanged *I*_NSE(intron)_. However, because the fraction of unspliced transcripts increases, the fraction of speckle-localized total transcripts greatly increases (9 of 14 = 64.3%), explaining the increase in *I*_NSE(exon)_. (**E**) GO analysis of speckle-enriched group A and group B genes, and non–speckle-enriched group C gene, performed using g:Profiler (*82*), with a background consisting of all group A, B, and C genes. GO terms in biological processes (BP) and cellular compartments (CC) were identified. “*N*” reports number of genes in (A) to (C). “*R*” reports Pearson’s correlation coefficient in (A). *P* values calculated with unpaired *t* test are reported above each violin plot in (B) and (C).

With these two refinements in place, we compared enrichment values under NT and Plad B treatment. We found a strong correlation between *I*_NSE(intron)_ values between NT and Plad B treatment (Pearson’s correlation coefficient, 0.84; Fig. 3A). The observed strong correlation suggests that at the pre-mRNA level, Plad B treatment overall has minimal impact on transcript speckle localization propensity, despite their increased abundance due to splicing inhibition, consistent with our observations on the fraction of unexcised introns (Fig. 2A). In contrast, we found that some genes exhibited large Plad B–dependent increase in *I*_NSE(exon)_ (Fig. 3A), suggesting that at the total transcript level, splicing inhibition differentially increases speckle enrichment of a subset of genes, consistent with the observed increase in speckle-localized fraction of total poly (A)^+^ RNAs (Fig. 2, B and C). To facilitate further analysis, we separated genes into three groups on the basis of their *I*_NSE(exon)_ values (Fig. 3A): group A genes with log_2_(*I*_NSE(exon)_) > 1, i.e., being >2-fold enriched under NT; group B genes with log_2_(*I*_NSE(exon)_) < 1 under NT, but log_2_(*I*_NSE(exon)_) > 1 upon Plad B treatment, i.e., showing Plad B–dependent speckle localization; and group C genes with log_2_(*I*_NSE(exon)_) < 1 under both conditions.

Comparison of *I*_NSE(exon)_ and *I*_NSE(intron)_ under NT and Plad B treatment conditions allowed us to infer the dynamics of transcript speckle localization under NT condition. Group A genes consistently demonstrate the highest *I*_NSE(exon)_ and *I*_NSE(intron)_ regardless of splicing inhibition (Fig. 3, B and C). We interpret it as an indication that group A transcripts are stably localized to speckles already under NT condition. Group B transcripts, whose *I*_NSE(exon)_ is similar to that of group C transcripts and much lower than that of group A transcripts (Fig. 3B), overall exhibit a significantly higher *I*_NSE(intron)_ compared to group C genes under NT condition (Fig. 3C). This feature of group B genes indicates that pre-mRNAs from group B genes are transiently enriched in speckles and that the spliced transcripts exit speckles upon rapid splicing, leading to the observed high *I*_NSE(intron)_ but low *I*_NSE(exon)_ under NT condition. Splicing inhibition increases the fraction of pre-mRNA among the totality of transcripts and causes an increase in *I*_NSE(exon)_ (Fig. 3D). Last, group C transcripts, which show low *I*_NSE(exon)_, also consistently show the lowest *I*_NSE(intron)_ regardless of Plad B treatment, suggesting that transcripts from this subset of genes are generally not localized to speckles throughout transcription or splicing. The insignificant change in *I*_NSE(exon)_ and *I*_NSE(intron)_ for group C transcripts upon Plad B treatment indicates that for transcripts with low speckle localization propensity, Plad B treatment is unlikely to increase their speckle localization.

In summary, genes classified in groups A, B, and C demonstrate different speckle localization propensity and dynamics: Group A transcripts are stably enriched in speckles; group B transcripts are transiently enriched in speckles at the pre-mRNA stage; and group C transcripts are not speckle enriched. Gene ontology (GO) analysis using the union of all three gene groups as background revealed that speckle-enriched group A and B genes are enriched in biological processes related to RNA metabolism and nucleus localization, whereas non–speckle-enriched group C genes are enriched in cellular compartments of extracellular organelle, membrane, cell periphery, and endoplasmic reticulum (Fig. 3E).

### Transcript speckle enrichment is positively correlated with localization of genes relative to speckles

**Fig. 4.**
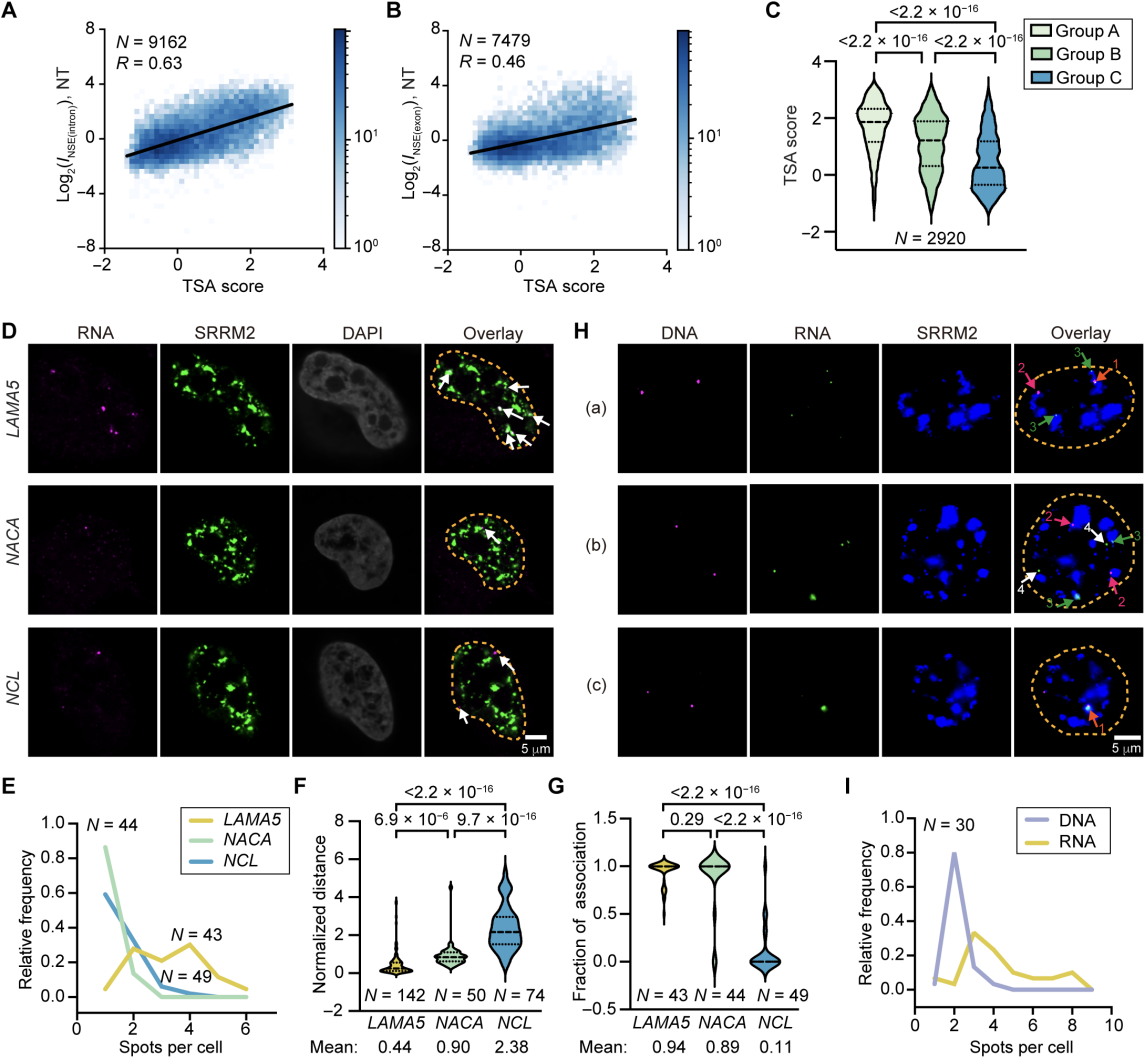
Correlation between RNA nuclear speckle enrichment and position of gene foci. (**A** and **B**) 2D histogram showing the correlation between *I*_NSE(intron)_ (A) or *I*_NSE(exon)_ (B) in HeLa cells and TSA score from TSA-seq in K562 cells. Genes with higher TSA scores are closer to speckles. (**C**) Violin plot comparing TSA scores for group A, B, and C genes. (**D**) RNA FISH images of unspliced transcripts of *LAMA5*, *NACA*, and *NCL* using intron targeting probes labeled with AF647 (magenta, also indicated with white arrows). Nuclear speckles were immunostained with AF488 (green). Nuclei were stained with DAPI (gray). (**E**) Histogram showing the distribution of the number of RNA foci per cell. (**F**) Normalized distance between each RNA focus and the nearest speckle, defined as the distance between their centers divided by the sum of their radii. A normalized distance of ~1 indicates that an RNA focus is localized to the periphery of a speckle. (**G**) Fraction of speckle-associated RNA foci using normalized distance <1.2 as a threshold. (**H**) (a to c) Combined RNA FISH (labeled with CF568, green) and GOLD FISH (labeled with AF647, magenta) detection of *LAMA5* transcripts and corresponding gene loci, respectively. Arrows with different labels indicate different DNA/RNA localizations: (1) speckle-associated colocalized RNA and DNA foci; (2) speckle-associated DNA foci without colocalized RNA foci; (3) speckle-associated RNA foci without colocalized DNA foci; and (4) RNA foci that are not associated with a DNA focus nor localized to speckles. (**I**) Histogram showing the distribution of the number of RNA foci and DNA foci per cell. “*N*” reports number of genes in (A) to (C) and number of cells in (E) to (I), collected from three biological replicates. *P* values calculated with unpaired *t* tests are reported above each violin plot in (C), (F), and (G).

Previous studies suggested that actively transcribed gene foci tend to be associated with nuclear speckles (*2*, *3*, *9*). We therefore analyzed the correlation between transcript speckle enrichment and the proximity of the gene foci to nuclear speckles, measured with tyramide signal amplification sequencing (TSA-seq) (*13*). TSA-seq labels gene foci in proximity of an immunostained nuclear compartment (nuclear speckles in this case) by tyramide free radicals generated by horseradish peroxidase. We found a positive correlation between TSA score and both *I*_NSE(intron)_ (Pearson’s correlation coefficient, 0.61 to 0.63; Fig. 4A and fig. S5A) and *I*_NSE(exon)_ (Pearson’s correlation coefficient, 0.46 to 0.49; Fig. 4B and fig. S5A). This suggests that transcripts from speckle-proximal gene foci tend to be more enriched in nuclear speckles, consistent with previous findings (*5*). The correlation with *I*_NSE(intron)_ is higher than that with *I*_NSE(exon)_; this supports the rationale that *I*_NSE(intron)_ reflects speckle enrichment of pre-mRNAs, which are mainly around transcription sites associated with gene foci, whereas *I*_NSE(exon)_ reflects localization of total transcripts either at the transcription sites or away from them. Last, TSA scores from group A and B genes are significantly higher than group C genes (Fig. 4C), supporting that transcripts that are either stably or transiently speckle enriched both have their DNA foci localized closer to speckles.

To further validate the spatial relationship between speckle enrichment and active transcription sites, we imaged the intron regions of transcripts from *LAMA5* (from group A), *NACA* (from group B), and *NCL* (from group C) using RNA FISH (Fig. 4D). We detected one to two RNA foci for *NACA* and *NCL*, presumably corresponding to transcription sites. However, more RNA foci were detected for *LAMA5* (Fig. 4E), indicating that a fraction of them are likely not representing transcription sites. To quantify the degree of speckle association of these RNA foci, we calculated the normalized distance between each RNA focus and the nearest speckle, defined as the distance between their centers divided by the sum of their radii. The mean normalized distance of *LAMA5* is around 0.4, suggesting that *LAMA5* transcripts localize to and also largely overlap with speckles. The mean normalized distance of *NACA* foci is around 0.9, consistent with these foci being positioned at the surface of speckles. The mean normalized distance of *NCL* foci is around 2, suggesting that they are not speckle associated (Fig. 4, F and G). In summary, to the extent that these imaged genes are representative, group A RNA foci are speckle associated, yet not only at transcription sites; group B RNA foci are speckle associated and likely represent transcription sites; and group C RNA foci are not speckle associated and also likely represent transcription sites.

To further investigate the spatial relationship between *LAMA5* RNA foci (containing unspliced *LAMA5* transcripts) and the actual transcription sites, we used the genome oligopaint via local denaturation FISH (GOLD FISH) method to detect DNA foci (*37*) and combined it with RNA FISH to detect RNA foci (Fig. 4H). Overlapping intron RNA FISH and GOLD FISH signals would suggest the accumulation of RNAs at transcription sites associated with DNA foci. We confirmed that the addition of GOLD FISH DNA labeling does not compromise the RNA FISH signal (fig. S5, B and C). Consistent with intron RNA FISH alone, *LAMA5* transcripts exhibit more RNA foci per cell than DNA foci (Fig. 4I). In addition, only 40 ± 9% of speckle-associated *LAMA5* RNA foci have a DNA focus associated to the same nuclear speckle, supporting that *LAMA5* RNA localization to speckle is not always associated with transcription sites. In summary, costaining of speckle-enriched RNA with the DNA foci supports that speckle localization of group A transcripts can be both cotranscriptional and posttranscriptional.

### Transcript speckle enrichment is weakly correlated with RNA abundance

We next wondered whether transcript speckle enrichment is correlated with the transcript’s expression level or abundance. We compared *I*_NSE(exon)_ and *I*_NSE(intron)_ values with gene expression levels [measured by poly (A)^+^ RNA-seq], nuclear RNA abundance (estimated by nuclear RNA-seq), and nascent transcript abundance [measured by global run-on sequencing (GRO-seq)] (*38*). The three abundance measures showed insignificant correlation with *I*_NSE(exon)_ but weak correlation with *I*_NSE(intron)_ (fig. S6). These comparisons suggest that speckle enrichment of total transcripts from each gene is not correlated with gene expression level or transcription activity (Pearson’s correlation coefficient, −0.13–0.07). However, speckle enrichment of pre-mRNAs is weakly correlated with gene expression level or transcription activity (Pearson’s correlation coefficient, 0.22 to 0.34). Such correlations are also consistent with the previous claim that being closely associated with speckles may positively affect transcription (*9*). The loss of correlation with *I*_NSE(exon)_ indicates that posttranscriptional localization of RNA is likely to be decoupled from transcript level or transcription activity.

### Transcript speckle enrichment is related to splicing timing and efficiency

**Fig. 5.**
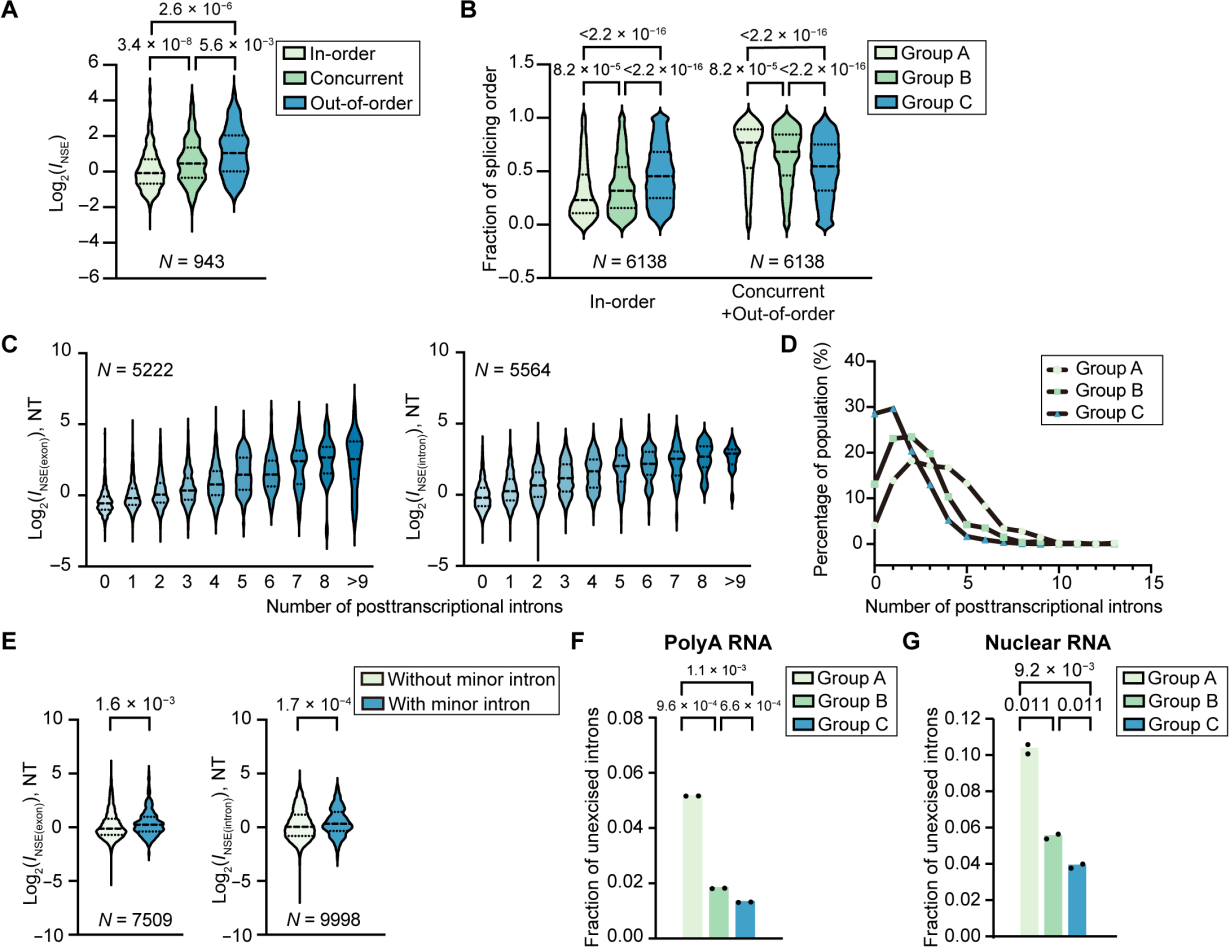
Transcript speckle enrichment is associated with splicing timing and efficiency in HeLa cells. (**A**) Violin plot comparing *I*_NSE_ values of in-order excised introns, concurrently excised introns, and out-of-order excised introns, as classified by CoLa-seq (*39*). (**B**) Fraction of in-order excised introns and not-in-order excised introns (concurrently excised introns and out-of-order excised introns) in group A, B, and C genes. (**C**) Violin plot comparing *I*_NSE(exon)_ or *I*_NSE(intron)_ values of transcripts containing different numbers of posttranscriptionally excised introns, as characterized by nanopore RNA-seq (*40*). (**D**) Histogram showing the distribution of posttranscriptionally excised intron number for group A, B, and C genes. (**E**) Violin plot comparing *I*_NSE(exon)_ or *I*_NSE(intron)_ values of transcripts containing minor splice sites and those without (*42*). In (A) to (E), *P* values calculated with unpaired *t* tests are reported above each violin plot. “*N*” reports the number of introns or genes. (**F** and **G**) Fraction of unexcised introns (computed as in Fig. 2A) for group A, B, and C genes under NT conditions at the poly (A)^+^ (F) and nuclear RNA level (G) using poly (A)^+^ RNA-seq and nuclear RNA-seq data. Each bar in (F) and (G) reports mean of the two RNA-seq replicates. *P* values were calculated with unpaired *t* tests.

We next analyzed whether speckle enrichment of transcripts is related to splicing timing measured in other studies. First, we used data from a study using cotranscriptional lariat sequencing (CoLa-seq) (*39*). By mapping intronic branch points, CoLa-seq reveals when an intron gets excised relative to its adjacent introns. Specifically, in-order excised (fast) represents the excision of an intron before transcription or excision of the downstream intron; out-of-order excised (slow) represents the excision of an intron after transcription and excision of one or more downstream introns; and concurrent excised (intermediate) reflects the excision of an intron around the same time as the downstream intron (*39*). We calculated *I*_NSE_ values for individual introns following the same analysis used to calculate *I*_NSE_ at the transcript level using normalization to *N*_-priAB_ and then compared these values to CoLa-seq data. We found that out-of-order and concurrently excised introns have significantly higher *I*_NSE_ values compared to in-order excised introns (Fig. 5A and fig. S7A). This suggests that the presence of introns with slow splicing kinetics correlates with high transcript speckle enrichment. In addition, group A genes contain significantly more introns with a small in-order excision fraction, followed by group B and group C genes, suggesting that group A genes are most enriched in slower excised introns (Fig. 5B).

Second, we compared *I*_NSE(exon)_ and *I*_NSE(intron)_ values of transcripts containing different numbers of posttranscriptional excised introns, as characterized using nanopore RNA-seq (*40*). We found that on average, a transcript’s speckle enrichment increases with the number of posttranscriptionally excised introns (Fig. 5C and fig. S7B). In addition, group A genes contain the most posttranscriptionally excised introns, followed by group B and group C genes, suggesting that group A genes have a higher contribution from posttranscriptional splicing (Fig. 5D).

Third, introns that use minor splice sites are known to be excised slower (*39*, *41*). We found that transcripts from genes containing introns using minor splice sites (*42*) are more enriched in speckles (Fig. 5E and fig. S7C).

Last, we compared the fraction of unexcised introns for group A, B, and C genes under NT condition using the poly (A)^+^ RNA-seq and nuclear RNA-seq data. Group A genes consistently demonstrate a 2.8- to 3.9-fold higher value compared to group B and C genes at the poly (A)^+^ RNA level (Fig. 5F and fig. S7D) and a 1.7- to 2.7-fold higher value compared to group B and C genes at the nuclear RNA level (Fig. 5G and fig. S7E). Collectively, these results indicate that speckle enrichment is associated with transcripts demonstrating slow splicing kinetics and containing posttranscriptionally excised introns under NT condition, supporting the view that nuclear speckles are involved in posttranscriptional splicing.

### Nuclear speckles facilitate splicing of speckle-enriched transcripts

**Fig. 6.**
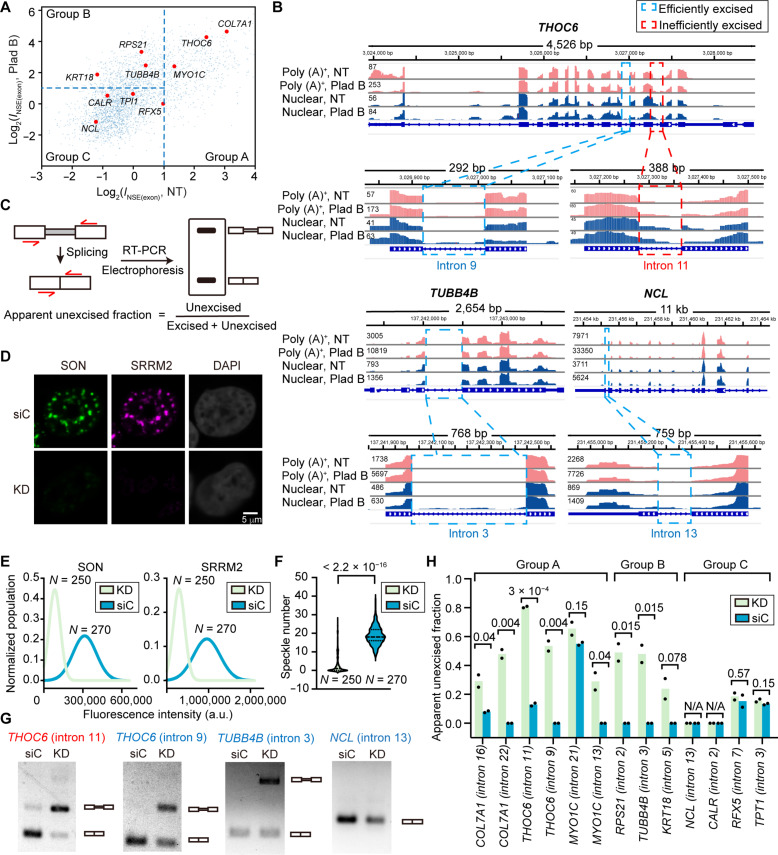
Nuclear speckles facilitate splicing of speckle-enriched transcripts. (**A**) Scatter plot showing randomly selected genes from group A, B, and C genes and corresponding *I*_NSE(exon)_ under NT and Plad B treatment conditions in HeLa cells. (**B**) Genome tracks showing poly (A)^+^ RNA-seq (pink) and nuclear RNA-seq (blue) under NT and Plad B treatment conditions for selected genes: *THOC6* (group A), *TUBB4B* (group B), and *NCL* (group C). Selected efficiently excised or inefficiently excised introns are highlighted in cyan and red boxes, respectively. (**C**) Schematic description of the RT-PCR assay. After reverse transcription of extracted total RNA, primers located on two adjacent exons of selected introns were used for amplification, and the PCR products were analyzed by electrophoresis. (**D**) Representative immunofluorescence images showing nuclear speckles upon *SON*/*SRRM2* double knockdown (KD) and treated with control siRNA (siC). SON (green) and SRRM2 (magenta) were stained with AF488 and CF568 respectively. Nuclei were stained with DAPI (gray). (**E**) Histogram of SON and SRRM2 fluorescence intensity distribution under KD and siC treatment. (**F**) Violin plot showing the number of speckles per cell for KD and siC treatment. Nuclear speckles were identified by a user-defined threshold of the total intensity of the SON and SRRM2 signals, applied to both knockdown and control samples. (**G**) Representative electrophoresis analysis of RT-PCR products from *THOC6*, *TUBB4B*, and *NCL* upon KD and siC treatment. (**H**) Apparent unexcised fractions of selected introns were calculated by ratios of the intensity of the unexcised band and the sum of the unexcised band and excised band. Each bar reports the mean unexcised fraction from two biological replicates. “*N*” reports the total number of cells included in each dataset in (E) and (F). *P* values calculated with unpaired *t* tests are reported above each violin plot or bar in (F) and (H).

To test whether nuclear speckles facilitate splicing of speckle-enriched transcripts, we randomly chose a few genes from groups A, B, and C (Fig. 6A). For each of these selected genes, we then picked introns that are either inefficiently excised [showing intronic reads in poly (A)^+^ RNA-seq or nuclear RNA-seq] or efficiently excised (not showing intronic reads) (Fig. 6B and fig. S8A). Using primers flanking the selected introns, we performed reverse transcription polymerase chain reaction (RT-PCR) analysis on cells that underwent either mock treatment [using control small interfering RNA (siRNA)] or speckle disruption by double siRNA knockdown of the scaffold proteins SON and SRRM2 (Fig. 6C). Upon double knockdown, immunostaining revealed a 60 to 65% decrease in both SON signal and SRRM2 signal (Fig. 6, D and E). The average number of speckles per cell dropped from 18.8 to 3.3 (Fig. 6F), indicating efficient speckle disruption. Also, the moderately speckle-enriched protein SRSF1 appeared to be uniformly distributed in the nucleoplasm upon speckle disruption (fig. S8B).

Under mock treatment, the RT-PCR assay confirmed the RNA-seq data, showing that introns containing mapped reads are inefficiently excised to various extents, whereas the rest of the introns are nearly fully removed (Fig. 6, G and H, and fig. S8C). *SON*/*SRRM2* double knockdown significantly affected the removal of eight of nine tested introns in group A and B transcripts but none in group C transcripts (Fig. 6H).

To ensure that the observed splicing impact of *SON/SRRM2* double knockdown is due to the disruption of nuclear speckles and not due to the reduced levels of the two proteins, we disrupted nuclear speckles by overexpressing a Cdc2-like kinase (*CLK1*) as demonstrated previously (*43*) (fig. S9, A to C). We again performed RT-PCR on select introns from group A, B, and C genes. The efficiency of speckle disruption by *CLK1* overexpression was lower than that of *SON/SRRM2* double knockdown (fig. S9C). Nevertheless, consistent with our earlier results, we observed that the excision of introns from group A and B transcripts is affected by *CLK1* overexpression but not the excision of introns from group C transcripts (fig. S9, D and E).

Collectively, these results suggest that nuclear speckles do not enhance splicing of all genes, but only of the subset of speckle-enriched transcripts (group A and B). This is consistent with the observation that group C transcripts are not speckle enriched (Fig. 3A). In addition, the sensitivity of group B transcript splicing to speckle disruption provides further evidence that pre-mRNAs of group B transcripts are transiently speckle associated under normal conditions and that their splicing is facilitated by speckles (Fig. 3, A and D). The alternative hypothesis that group B spliced transcripts are not speckle localized but become localized because of the splicing inhibition is not supported by the data because speckle disruption is unlikely to affect transcripts that do not localize there. These results also suggest that speckle-facilitated splicing may occur both cotranscriptionally (for group B transcripts, which are more transiently enriched in speckles at the pre-mRNA stage cotranscriptionally) and posttranscriptionally (for group A genes, which demonstrate features associated with posttranscriptional localization).

### A regression model predicts RNA sequence features associated with speckle enrichment

We next sought to identify cis-factors that contribute to nuclear speckle enrichment, focusing on *I*_NSE(exon)_ under NT and *I*_NSE(intron)_ under Plad B. On the basis of the above analyses, we reasoned that high *I*_NSE(exon)_ under NT reflects stably speckle-enriched total transcripts, whereas high *I*_NSE(intron)_ under Plad B treatment reflects either transiently or stably speckle-enriched pre-mRNAs. Consistent with a previous study with APEX-seq (*5*), we found that both quantities demonstrate a consistent positive dependence on intronic (Pearson’s correlation coefficient, 0.54 to 0.63) and exonic GC content (Pearson’s correlation coefficient, 0.48 to 0.55) and a negative dependence on the average intron length (Pearson’s correlation coefficient, −0.75 to −0.45) and total gene length (Pearson’s correlation coefficient, −0.69 to −0.23), while the dependence on exon length is less obvious (fig. S10, A to C).

**Fig. 7.**
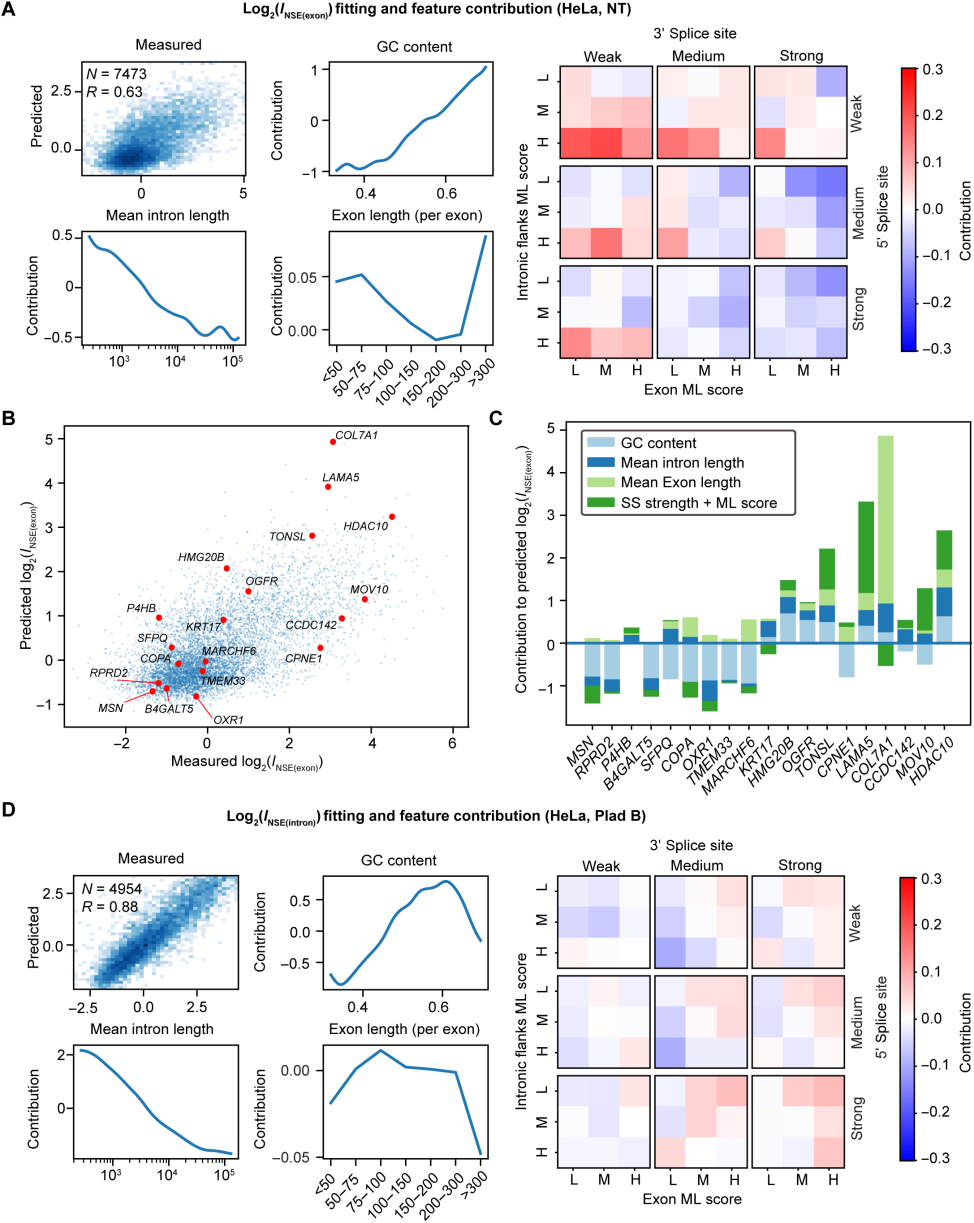
Regression model predicts RNA sequence features associated with speckle enrichment. Input parameters and other related details of the regression model are described in the main text. (**A**) The exon-centric regression model reveals contributions from GC content, mean intron length, individual exon length, and a combination of splice site strength, exonic ML score, and flanking intronic ML score to the transcript speckle enrichment *I*_NSE(exon)_ values under NT in HeLa cells. (**B**) Predicted *I*_NSE(exon)_ values using the regression model on randomly selected genes are consistent with the measured *I*_NSE(exon)_ values from ARTR-seq. (**C**) The relative contribution from each parameter on selected genes. (**D**) The exon-centric regression model reveals contributions from GC content, mean intron length, individual exon length, and a combination of splice site strength, exonic ML score, and flanking intronic ML score to the transcript speckle enrichment *I*_NSE(intron)_ values under Plad B treatment in HeLa cells. “*N*” reports the number of genes in the regression analysis, and “*R*” reports Pearson’s correlation coefficient.

To obtain a more detailed understanding of the relevant RNA sequence features, we used a regression model [generalized additive model (GAM)] and fit it to the measured *I*_NSE_ values. The choice of input features to the model was based on the dependencies observed above and the correlation with splicing efficiency. Specifically, we included a gene’s GC content and its mean intron length as input features to the model. Because speckle enrichment demonstrates a similar correlation with intronic and exonic GC content (fig. S10, A to C), we did not separate these two in the regression model. Moreover, while intron length and gene length are strongly correlated with each other (Pearson’s correlation coefficient, 0.74; fig. S10D), intron length demonstrates a higher correlation with *I*_NSE(exon)_ and with *I*_NSE(intron)_ (fig. S10, A to C). We therefore chose to use mean intron length as input in our model. In addition to these two gene-level input features, we also included several splicing-related features for each internal exon. These include the exon length, the strength of its flanking acceptor (3′) and donor (5′) splice sites, as determined using MaxEnt (*44*), and a machine learning (ML) score of the exon sequence and of the flanking intronic sequences. This ML score is computed using a model trained on splicing assay data (*45*). It is high for sequences that are recognized as exons (such as those enriched in binding sites of SR family proteins) and low for sequences that are recognized as introns (such as those enriched in binding sites of hnRNP family proteins). Each of the latter four features (3′ splice site strength, 5′ splice site strength, exonic ML score, and intronic ML score) is quantile binned into one of three bins, allowing to categorize each exon into one of 3^4^ = 81 possible combinations. To summarize, the input to the model consists of the gene’s GC content, its mean intron length, and a list of its internal exon features, each described by its length and a categorical value corresponding to its splice site strengths and ML scores. To arrive at its prediction, the model scores each of these separately and outputs the total score. Using these features, the regression model achieved an excellent fit to the measured enrichment values (Fig. 7).

### Speckle-enriched RNAs under NT condition demonstrate sequence features associated with inefficient splicing

We next interpreted the regression model to understand how the various features correlate with transcript speckle enrichment under NT condition (Fig. 7 and fig. S11). Consistent with our previous correlation analysis, when analyzing *I*_NSE(exon)_ under NT, we found that high GC content and low mean intron length contribute significantly to speckle enrichment. The model revealed that short exons (<75 nt) contribute to speckle enrichment, possibly related to the fact that they do not splice efficiently (*46*, *47*). In addition, exons with a combination of weak splice sites and a high ML score for the flanking intronic sequences (suggesting those intronic sequences are not well defined) are positively correlated with speckle enrichment. A mild contribution from a low ML score for the exon sequence (suggesting an exon that is not well defined) was also observed. The same effects were consistently revealed in both HeLa and HepG2 cells (Fig. 7A and fig. S11A).

We further tested an alternative “intron-centric” regression model (fig. S11, B and C), in which internal introns are categorized instead of internal exons. Specifically, for each internal intron, we used the strength of its flanking 5′ and 3′ splice sites, its ML score, and the ML score of the flanking upstream and downstream exons. These features were binned and combined as above, allowing us to label each internal intron with 1 of 81 possible combinations. The remaining features (GC content, mean intron length, and exon lengths) were kept the same. The sequence features identified by this model are largely consistent with the previous exon-centric model. Namely, high GC content, short mean intron length, short exon lengths, and a combination of weak splice sites and high intronic ML scores are all positively correlated with speckle enrichment, though the dependence on exonic ML score was not obvious in the intron-centric model.

To demonstrate the regression model’s prediction process, we randomly selected several genes and used the model to predict their speckle enrichment from sequence features. In agreement with the model’s good fit, the predicted values are well correlated with the *I*_NSE(exon)_ values experimentally measured by ARTR-seq (Fig. 7B). The predictions are not dominated by any one feature; instead, each sequence feature (GC content, mean intron length, exon lengths, and splice site strengths with ML scores) can be the major contributing factor in a transcript-dependent manner (Fig. 7C). A similar transcript-dependent feature contribution was also observed for predicting splicing timing (*39*).

In summary, the regression analysis on *I*_NSE(exon)_ values under NT reveals features that distinguish group A transcripts from group B and C transcripts under normal conditions. Features including high GC content, short introns and exons, and a combination of weak splice sites with high intronic ML scores all contribute to speckle enrichment. Similar features (namely, high intronic GC content, short intron length, weak splice sites, and enrichment of intronic SR protein binding motifs) were previously reported for retained introns (*48*–*50*). These results suggest that transcripts that are difficult to splice fully (because of the presence of weak splice sites or suboptimal cis-factors within exons or introns) are preferentially enriched in nuclear speckles, likely posttranscriptionally. These results are also in line with the hypothesis that nuclear speckles participate in posttranscriptional splicing.

### Speckle-localized pre-mRNAs demonstrate sequence features associated with efficient splicing

Performing the same analysis on *I*_NSE(intron)_ under NT revealed a similar dependence on GC content and intron length as in the previous *I*_NSE(exon)_ analysis. However, features associated with splicing are strongly diminished (fig. S11D). Because *I*_NSE(intron)_ reflects pre-mRNA speckle enrichment, the disappearance of splicing-related features indicates that speckle-enriched pre-mRNAs may not exhibit the same splicing-related features as speckle-enriched total transcripts.

Because splicing inhibition increases the contribution of pre-mRNA, we repeated the same regression analysis on *I*_NSE(intron)_ under Plad B treatment (Fig. 7D). GC content and intron length dependence were robustly identified. In contrast, this analysis revealed different splicing-related features, which are largely opposite of those identified in the *I*_NSE(exon)_ analysis (Fig. 7A and fig. S11A). Specifically, speckle-enriched genes exhibit a moderate preference for strong 5′ and 3′ splice sites in combination with a high exonic ML score. These features together with a preference of high GC content and short intron length are more associated with speckle-localized pre-mRNAs. As strong splice sites and strong exonic ML scores are features associated with efficient splicing (*45*), this correlation indicates that these pre-mRNA transcripts undergo efficient splicing.

Independent analysis of GC content, intron length, and splicing-related features in group A, B, and C transcripts further confirmed that speckle-localized group A and B transcripts have distinct features (fig. S12). Group A transcripts have the highest GC content, shortest average intron length, and a preference for a combination of weak splice sites and high intronic ML scores. Group B transcripts have an intermediate GC content, an intermediate average intron length, and a preference for the combination of strong splice sites with high exonic ML scores. Last, group C transcripts have the lowest GC content, longest average intron length, and a preference for low intronic and exonic ML scores independent of splice site strength.

### Transcripts with up-regulated intron retention during heat shock are enriched in nuclear speckles

Membraneless organelles play important roles in stress response (*51*, *52*). For example, upon stress, translationally paused mRNA can be temporarily sequestered to cytoplasmic stress granules (*51*). Our results demonstrate that nuclear speckles accommodate inefficiently spliced transcripts (group A genes). Because intron retention is known to regulate gene expression through diverse mechanisms (*48*, *50*) including nuclear detention (*50*, *53*–*55*), we wondered whether cells use speckles to respond to stress.

**Fig. 8.**
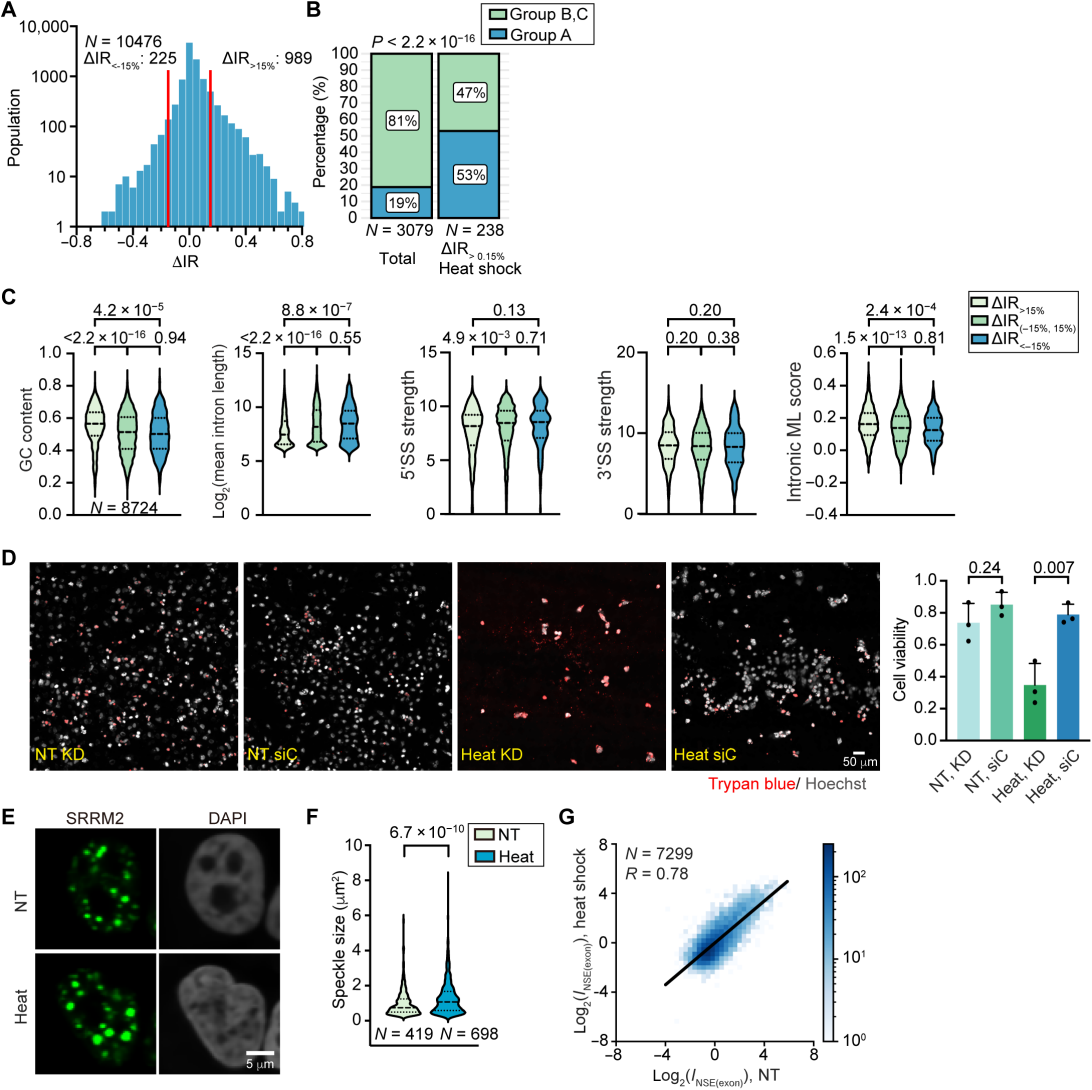
Functional implication of nuclear speckle under heat shock. (**A**) IRFinder analysis showing heat shock–induced up-regulation of intron retention. The number of intron retention events with more than 15% increase (∆IR_>15%_) or decrease (∆IR_<-15%_) are labeled. (**B**) Percentage of group A genes and group B and C genes without and with taking the subset of genes containing ∆IR_>15%_ introns. *P* value: Fisher’s exact test. (**C**) Violin plot showing the group A–like sequence feature associated with three groups of introns (ΔIR_>15%_, ΔIR_(−15%, 15%)_, ΔIR_<-15%_). The GC content, intron length, splice site strength, and intronic ML score are compared for three groups of introns. (**D**) Viability of HeLa cells under nuclear speckle disruption via *SON/SRRM2* double KD or under siC upon heat shock stress or NT. Hoechst staining reflects the total cell population, whereas trypan blue stains dead cells. Cell viability was calculated as one minus the fraction of dead cells (1 − *N*_Dead_/*N*_Total_), where *N*_Dead_ and *N*_Total_ are the number of dead cells and total cell number, respectively. *P* values calculated with unpaired *t* test are reported above each violin plot and box plot. Error bars report SD from three biological replicates (in black dots). (**E**) Immunofluorescence image of nuclear speckles under NT and heat shock conditions. (**F**) Violin plot showing the speckle size in heat shock compared to NT. (**G**) 2D histogram showing the correlation between *I*_NSE(exon)_ values under heat shock and NT. “*N*” reports the number of introns in (A) and (C), the number of genes in (B) and (G), and the number of speckles in (F).

To address this question, we used heat shock as an example. We stressed the cells at 43°C for 2 hours and performed poly (A)^+^ RNA-seq. Consistent with previous results using mouse fibroblasts (*56*), we observed an overall up-regulation of intron retention in poly (A)^+^ RNA-seq upon heat shock as identified by IRFinder (*49*), with >4-fold more up-regulated than down-regulated intron retention events (Fig. 8A). With the exception of one gene (*HSPE1*), none of the classic heat-responsive heat shock proteins (*57*, *58*), including those belonging to the heat shock protein family A (HSPA) (HSP70), HSPB (small HSP), HSPC (HSP90), HSPH (HSP110), and DNAJA/DNAJB (HSP40) families, exhibits heat shock–induced intron retention increase. Consistently, GO enrichment analysis of genes containing introns demonstrating >15% increased retention upon heat shock (ΔIR_>15%_) did not identify any heat response–related term, no matter whether the analysis background was chosen as the whole genome or as all expressed genes. These results suggest that intron retention may be used to negatively regulate the expression of non–heat stress–related genes, providing a survival benefit.

We next considered a possible role for speckle localization in heat shock–induced intron retention. We found that 53% of ΔIR_>15%_ genes are in group A, although group A genes constitute only 19% of all classified genes (Fisher’s exact test *P* < 2.2 × 10^−16^; Fig. 8B). To further support this correlation, we inspected the sequence features exhibited by heat shock–induced retained introns. We found that ΔIR_>15%_ introns exhibit stronger group A–like sequence features than other introns, including significantly higher GC content, shorter length, weaker 5′ splice site, and stronger intronic ML score (Fig. 8C). In summary, transcript speckle localization is strongly correlated with heat shock–induced intron retention. Further supporting a role of speckles in heat shock response, we found that cells with *SON*/*SRRM2* double knockdown demonstrate reduced viability upon heat shock compared to the ones treated with the control siRNA (Fig. 8D).

We next explored whether heat shock induces any changes in speckle morphology or in transcript speckle enrichment. We observed a significant increase in speckle size upon heat shock (Fig. 8, E and F). This is consistent with a previous study illustrating that transcripts with heat shock–induced intron retention are retained in the nucleus (*56*). However, when we performed ARTR-seq on stressed cells, *I*_NSE(exon)_ values were strongly correlated with those under NT (Pearson’s correlation coefficient, 0.78; Fig. 8G). This indicates that, unlike what was observed with the splicing inhibition perturbation (Fig. 3A), heat shock does not significantly affect transcript speckle enrichment. Consistently, when applying the regression analysis on *I*_NSE(exon)_ values under heat shock, we found the same sequence features as we identified under unstressed condition (fig. S13). In summary, the increase in speckle size upon heat shock seems to be driven mainly by the increased abundance of transcripts containing retained introns and not by changes in transcript speckle enrichment. Collectively, these analyses suggest that speckle-enriched group A genes tend to be more sensitive to splicing perturbation, likely because of the presence of weak splicing-related sequence features, and point to speckles playing a role in accommodating transcripts with heat shock–induced intron retention.

## DISCUSSION

In this work, we broadened the applicability of our recently developed ARTR-seq method to transcriptomically map nuclear speckles. While ARTR-seq was originally developed as a method to identify direct protein binding sites, given the high density of speckle-localized RNAs, it is also robust in capturing transcripts in the vicinity of the antibody anchoring point. The captured RNAs might be direct or indirect binding targets of SON and SRRM2. Distinguishing the two possibilities is beyond the focus of the current work, and our results are valid in either case. Compared to a related transcriptomic analysis on nuclear speckles using APEX-seq (*5*), our study provides an alternative approach with several advantages. First, our approach can better preserve the integrity of nuclear speckles by directly targeting endogenous speckle marker proteins and by avoiding potential cell stresses caused during sample treatment, such as using hydrogen peroxide. Second, compared to using SRSF1 and SRSF7 as speckle-targeting proteins (*5*), we target the most speckle-enriched scaffold proteins SON and SRRM2 (*29*), thereby increasing targeting specificity to nuclear speckles. Third, the method is very flexible and can be readily adapted to other marker proteins of interest without requiring the generation of fused proteins. Last, in situ reverse transcription avoids the use of diffusive radicals, potentially increasing the localization accuracy for studying membraneless organelles compared to APEX-seq.

We calculated *I*_NSE_ values by normalizing to a dataset obtained using the same ARTR-seq method but without primary antibody. By doing so, we eliminated potential sequence biases in ARTR-seq and obtained a more accurate *I*_NSE_ estimate, as verified by RNA FISH imaging. We also considered an alternative normalization method, using standard nuclear RNA-seq, unrelated to ARTR-seq, which reflects the nuclear abundance of each RNA species (fig. S14). Although *I*_NSE,SON_ and *I*_NSE,SRRM2_ are also highly correlated using this alternative normalization method (Pearson’s correlation coefficient, 0.81 to 0.89; fig. S14B), the quantitative agreement with RNA FISH imaging results is worse (Pearson’s correlation coefficient, 0.28; fig. S14C), likely because standard nuclear RNA-seq cannot eliminate the sequence bias in ARTR-seq because of the different sample preparation. Another possible normalization method is against ARTR-seq targeting an ideally uniformly distributed protein. However, such a normalization method may also introduce biases due to potential interacting RNAs in the normalization sample. Imaging validation should be performed to evaluate any alternative normalization method. Last, while we mainly use *I*_NSE,SON_ for our analyses, key results are reproduced using *I*_NSE,SRRM2_ (fig. S15).

**Fig. 9.**
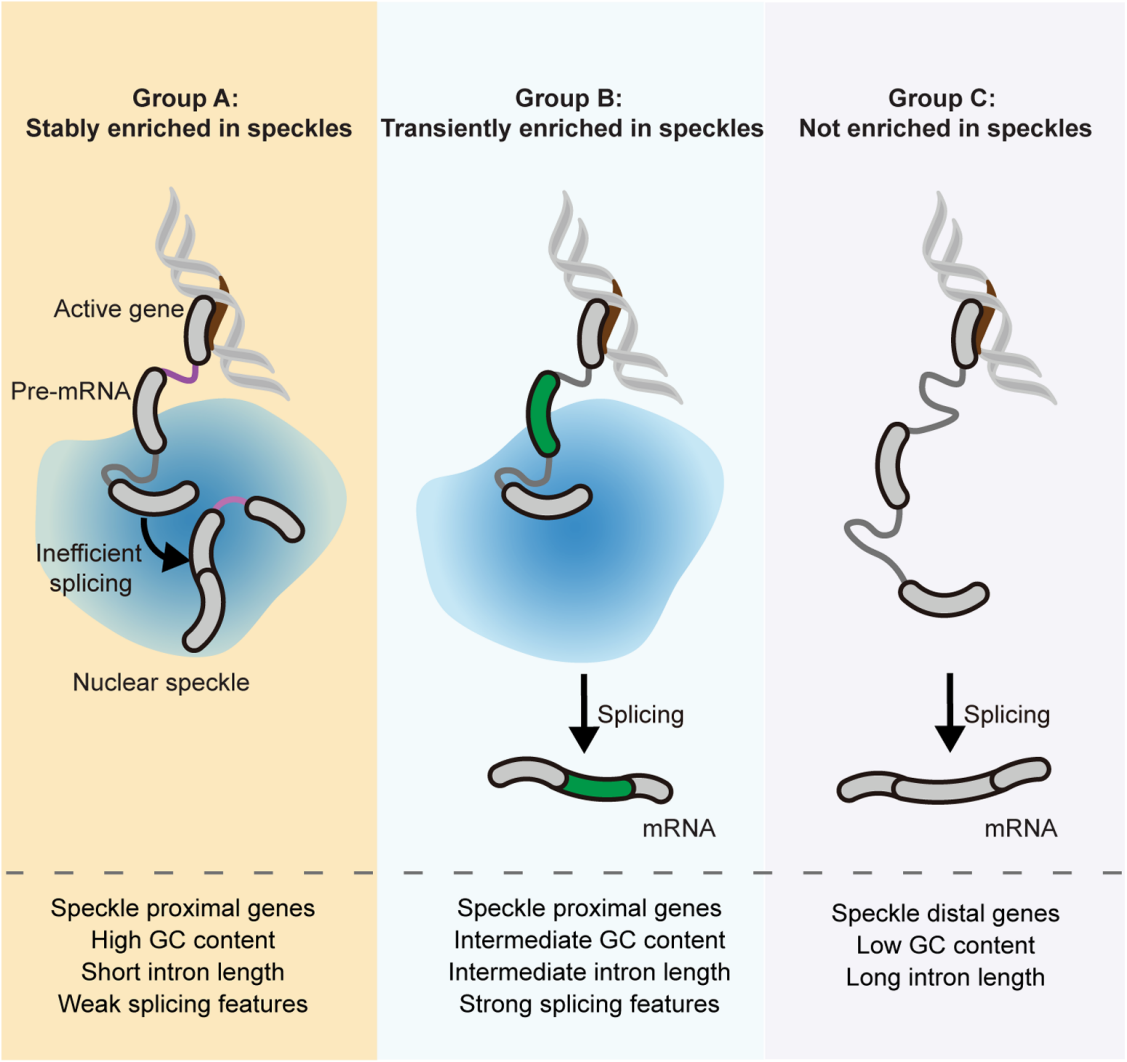
Classification of gene groups based on nuclear speckle localization of transcripts. Summary of the three gene groups in terms of nuclear speckle localization of transcripts, splicing features, gene position relative to speckles, and sequence features. See text for details.

Our results provide strong evidence (through disruption of nuclear speckles) for the role of nuclear speckles in splicing and demonstrate that this role differs between the three gene groups (Fig. 9). Transcripts from group A genes are speckle enriched at the pre-mRNA stage (high *I*_NSE(intron)_ under NT), likely cotranscriptionally, and remain enriched posttranscriptionally (high *I*_NSE(exon)_ under NT), presumably because of the presence of one or more slowly excised or retained introns. Transcripts from group B genes are also speckle enriched at the pre-mRNA stage (high *I*_NSE(intron)_ under NT) but exit speckles after splicing (low *I*_NSE(exon)_ under NT). Last, transcripts from group C genes are not enriched in speckles (low *I*_NSE(intron)_ and low *I*_NSE(exon)_ under NT). Disruption of nuclear speckles affects the splicing efficiency of both group A and B transcripts but not group C transcripts. Collectively, our data indicate that nuclear speckles facilitate both co- and posttranscriptional splicing for a subset of genes and present mechanistic insights into the intricate relationship between nuclear speckles and the splicing process.

Our data support the previous observation that not all actively transcribed genes are speckle associated (*9*) and reveal a tight interplay between genomic organization, RNA localization to nuclear speckle, and sequence features. Consistent with previous results from APEX-seq (*5*), we found that gene proximity to nuclear speckles is moderately correlated with total transcript speckle enrichment and more strongly to the pre-mRNA speckle enrichment. Consistently, group A and B gene foci are both closer to nuclear speckles compared to group C genes. RNA FISH imaging targeting intron sequences confirms that the transcription sites of group B genes are associated with speckles but not those of group C genes. Group A transcripts, however, demonstrate more RNA foci compared to DNA foci revealed by DNA/RNA costaining, supporting that group A transcript localization to speckle can occur at the transcription sites, likely cotranscriptionally, or away from transcription sites, likely posttranscriptionally. We also found that some group C genes, such as *CALR* and *TPI1*, have a high TSA score, suggesting that gene position alone cannot explain transcript localization to speckles. Using regression analysis, we further uncovered sequence features that positively contribute to RNA localization to nuclear speckles and found that having short GC-rich introns is associated with higher transcript speckle enrichment, both at the total transcript and at the pre-mRNA levels. Genes containing these introns are organized in the interior region of the nucleus (*59*). Therefore, the association of these features with group A and B genes might be related to genome organization. Namely, it is possible that gene position affects transcript speckle localization. Alternatively, cis-elements leading to transcript speckle localization facilitate the recruitment of speckles to the transcription sites or the movement of gene foci toward nuclear speckles.

Our regression analysis also identified a correlation between transcript speckle localization and splicing-related sequence cis-elements (Fig. 7), suggesting a model in which nuclear speckles coordinate splicing both co- and posttranscriptionally. Features such as weak splice sites in combination with intronic SR protein binding motifs (reflected by high intronic ML score) appear in group A genes (whose total transcripts are speckle enriched), supporting the role of nuclear speckles as a processing site for slowly excised or retained introns. In contrast, when considering speckle enrichment at the pre-mRNA level, the regression analysis identified some splicing-favored elements, such as strong splice sites in combination with exonic SR protein binding motifs (reflected by high exonic ML score), as being associated with higher pre-mRNA speckle enrichment. These correlations support the hypothesis that pre-mRNAs with these features from group B genes, which are globally more efficiently spliced than group A transcripts, are cotranscriptionally localized to speckles and exit speckles upon splicing. Group A transcripts, which are more enriched in slowly excised introns and globally contain a higher fraction of unexcised introns than group B transcripts, are further retained and spliced in nuclear speckles posttranscriptionally.

While the exact mechanisms underlying the correlation between RNA cis-elements and speckle localization remain to be investigated, we hypothesize the following factors: (i) High GC content in the speckle-associated group A and B transcripts may naturally lead to a higher propensity to partition into phase-separated domains (*60*, *61*). (ii) Similarly, the presence of more SR protein binding motifs associated with group A and B transcripts may contribute to speckle localization given the speckle enrichment of many SR proteins (*29*). (iii) Splicing promoting cis-sequence features in group B transcripts may facilitate spliceosome assembly on these pre-mRNAs, which increases their speckle localization given the known speckle enrichment of spliceosomal components (*2*, *29*, *62*).

Our model of nuclear speckles participating in both co- and posttranscriptional splicing is consistent with earlier observations using imaging. For example, a posttranscriptionally excised intron (intron 24 of *COL1A1*) and a retained intron (mutation-containing intron 26 of *COL1A1*) were observed to accumulate in nuclear speckles (*10*, *63*). These imaging experiments also revealed intraspeckle positional differences between co- and posttranscriptionally excised introns in *COL1A1*: Cotranscriptionally excised introns stay at the periphery of speckles with the gene foci outside speckles, whereas posttranscriptionally excised introns and retained introns are distributed throughout the speckle. Consistently, we also observed that *LAMA5* transcripts (group A) localize more to the interior region of speckles compared to *NACA* transcripts (group B), which localize to the speckle periphery (Fig. 4E). Therefore, it is possible that pre-mRNA transcripts from group B genes and inefficiently spliced transcripts from group A genes have different intraspeckle localizations. However, further investigations are needed to clarify the functions of the speckle core and outer shell in coordinating co- and posttranscriptional splicing.

Repression of genes that are not directly needed for stress response can occur at the splicing level through intron retention (*50*, *53*–*55*). Our study unveils a compelling function of nuclear speckles in the regulation of intron retention under heat shock stress. We found that under heat shock, a global increase in intron retention is correlated with an increased speckle size. Moreover, group A genes (whose transcripts are speckle enriched) are over-represented in genes demonstrating heat shock–induced increase in intron retention. Consistently, introns retained under heat shock demonstrate group A–like sequence features associated with speckle localization. These correlations suggest that group A transcripts are more sensitive to heat stress–induced changes in splicing factors, likely because of the presence of weak splicing-related sequence features, demonstrate up-regulated intron retention, and accumulate in speckles. In other words, cells use the shared sequence features between retained introns and speckle-localized group A transcripts as one way to negatively regulate gene expression at the splicing level with speckles as a storage site for transcripts with retained introns. Moreover, the enrichment of spliceosomal components in speckles (*2*, *3*) may facilitate splicing upon recovery from stress. Further investigations are needed to fully elaborate the relationship between stress, intron retention, and nuclear speckle localization.

Last, we discuss a few limitations of the current study. ARTR-seq tends to provide relatively short reads, preventing confident isoform-level analysis. It also produces uneven read coverage within transcripts, complicating attempts to identify intramolecular speckle localization differences. These limitations can be partially attributed to the accessible range (considering the finite linker length between pAG and RTase, possible RNA folding, and protein binding in certain regions) or the sequence bias of the RTase. While they do not affect our current analysis, further optimization of the method can potentially overcome them, allowing for a more detailed analysis of the interaction between transcription, splicing, and nuclear speckles. In addition, formaldehyde fixation in ARTR-seq may affect protein phase separation behavior in certain cases (*64*). While this fixation method has been routinely applied in the study of nuclear speckles, other membraneless organelles may need an alternative fixation method. The built-in imaging step in ARTR-seq can help validate the morphology of membraneless organelles during sample preparation. While Plad B is commonly used as a splicing inhibitor, its effect is known to be sequence dependent (*65*, *66*). This may introduce biases to the categorization of group B genes and our analysis on identifying RNA cis-elements that contribute to pre-mRNA speckle localization. We observed that group C genes are more than twofold under-represented in the set of transcripts exhibiting large Plad B–induced intron retention increase (fig. S3, G and H). Therefore, it is possible that some genes currently categorized in group C also have transient association with speckles and belong in group B. Application of additional splicing inhibitors may generate a more complete list of group B genes. We do note, however, that our FISH and speckle disruption experiments provide orthogonal evidence (independent of splicing inhibition) that some group C transcripts are truly not speckle associated. Last, while we assume that most pre-mRNAs undergo splicing cotranscriptionally and interpret *I*_NSE(intron)_ under the NT condition to mostly reflect cotranscriptionally spliced pre-mRNAs, current ARTR-seq experiments cannot distinguish co- from posttranscriptional splicing. It is likely that a fraction of group B pre-mRNAs undergo rapid posttranscriptional splicing after transcription termination at nuclear speckles.

## MATERIALS AND METHODS

### Cell culture and treatment

HeLa human cervical cancer cells and HepG2 human hepatocellular carcinoma cells were cultured in high-glucose Dulbecco’s modified Eagle’s medium (DMEM, Gibco) supplemented with glucose (4.5 g/liter), 1 mM sodium pyruvate, penicillin/streptomycin solution (50 U/ml, Gibco) and 10% fetal bovine serum (Gibco). Mycoplasma contamination was regularly tested for both cell lines. For fluorescence imaging, HeLa cells were seeded at a density of 3 × 10^4^ cells in an eight-well imaging chamber (no. 1.5 cover glass, Cellvis) and grown overnight to 70 to 80% confluency. For HepG2 cells, chamber was coated with 100 μl of Matrigel matrix (Corning, 5 mg/ml) at 37°C for 1 hour before seeding the cells.

For splicing inhibition experiment, cells were treated with Plad B (100 nM, Cayman Chemical) at 37°C for 4 hours in DMEM. For heat shock, cells were incubated at 43°C for 2 hours before following experiments.

### Disruption of nuclear speckles

Speckle disruption by knocking down of *SON* and *SRRM2* in HeLa cells was performed using Lipofectamine RNAiMAX Transfection Reagent (Thermo Fisher Scientific). siRNAs were designed and purchased from Integrated DNA Technologies (IDT). The cells were sequentially transfected with siRNA (*SON*) and siRNA (*SRRM2*) with a 24-hour interval between each transfection with a final concentration of 5 nM. The cells were also transfected with the same concentration of control siRNA twice as a negative control. The cells were subsequently incubated at 37°C for an additional 48 hours before further experiments.

In speckle disruption by *CLK1* overexpression, HeLa cells were transiently transfected with 500 ng of *RFP*-*CLK1* plasmid DNA (*43*) (a gift of Y. Shav-Tal) using Lipofectamine 3000 Transfection Reagent (Thermo Fisher Scientific) following the manufacturer’s instructions. At 48 hours after transfection, cells were fixed for imaging experiments or proceeded with total RNA extraction for RT-PCR experiments.

### Poly (A)^+^ RNA-seq and nuclear RNA-seq

#### Nucleus isolation

HeLa cells or HepG2 cells were collected by centrifugation at 500*g* for 3 min and washed once with 1 ml of Dulbecco’s phosphate-buffered saline (DPBS). The cell pellet was resuspended in 200 μl of ice-cold lysis buffer [10 mM tris-HCl (pH = 7.5), 0.15% NP-40, 150 mM NaCl], and incubated on ice for 5 min. Then, the cell lysate was gently pipetted up over 500 μl of chilled sucrose cushion (24% RNase-free sucrose in lysis buffer) and centrifuged at 15,000*g* for 10 min at 4°C. The pellet was collected as nuclei.

#### RNA extraction

Total RNA from nuclei or whole cells was purified with TRIzol reagent (Thermo Fisher Scientific) according to the manufacturer’s instructions. A RiboMinus Eukaryote kit (Thermo Fisher Scientific) was used to remove rRNA from nucleus RNA. A Dynabeads mRNA DIRECT kit (Thermo Fisher Scientific) was used to extract poly (A)^+^ RNA from total RNA. The RNA concentration was measured by NanoDrop 8000 Spectrophotometer (Thermo Fisher Scientific).

#### RNA sequencing

RNA-seq libraries of rRNA-depleted nuclear RNA or poly (A)^+^ RNA were prepared with the SMARTer Stranded Total RNA-Seq Kit v2 (Takara) according to the manufacturer’s protocols. Sequencing was performed at the University of Chicago Genomics Facility on an Illumina NovaSeq 6000 platform in single-end mode with 100 bp.

### Reverse transcription–based RNA binding protein binding sites sequencing

ARTR-seq was performed according to the previously published procedure (*26*). Briefly, HeLa or HepG2 cells were fixed with 1.5% paraformaldehyde (PFA) for 10 min at room temperature, quenched with 125 mM glycine, and permeabilized with 0.5% Triton X-100 on ice for 10 min. Samples were blocked with UltraPure bovine serum albumin (BSA) (1 mg/ml, Thermo Fisher Scientific), stained with SON or SRRM antibodies at room temperature for 1 hour, and then stained with fluorophore-labeled secondary antibody at room temperature for 30 min. Samples were then incubated with pAG-RTase for an additional 30 min. A reverse transcription reaction mixture was prepared by mixing 2 μM adapter-RT primer (5′-AGACGTGTGCTCTTCC-GATCT-10 N-3′), 0.05 mM biotin-16-dUTP (deoxyuridine triphosphate) (Jena Bioscience), 0.05 mM biotin-16-dCTP (deoxycytidine triphosphate) (Jena Bioscience), 0.05 mM deoxythymidine triphosphate (Thermo Fisher Scientific), 0.05 mM dCTP (Thermo Fisher Scientific), 0.1 mM deoxyadenosine triphosphate (Thermo Fisher Scientific), 0.1 mM deoxyguanosine triphosphate (Thermo Fisher Scientific), RNaseOUT (1 U/μl, Thermo Fisher Scientific) in 50 μl of buffer of DPBS supplemented with 3 mM MgCl_2_. In situ reverse transcription was performed by adding RT reaction mixture to cells and incubating at 37°C for 30 min and then quenched by adding 20 mM EDTA and 10 mM EGTA. To check the success of in situ reverse transcription, cells were stained with biotin monoclonal antibody (BK-1/39, RRID:AB_10598675) conjugated with Alexa Fluor 488 (AF488, Thermo Fisher Scientific), and imaged by a Leica SP8 laser confocal microscope. The fluorescence intensity distribution on a line was quantified by Fiji (version 2.3.0) (*67*). After imaging, cells were digested with proteinase K (Thermo Fisher Scientific), and the nucleic acids, including the generated biotinylated cDNA, were recovered by phenol-chloroform extraction and concentrated by ethanol precipitation. RNA was digested with RNase H (NEB) and RNase A/T1 (Thermo Fisher Scientific) at 37°C for 1 hour, followed by biotinylated cDNA enrichment using Dynabeads MyOne Streptavidin C1 (Thermo Fisher Scientific). The 3′ cDNA adapter (5′Phos-8 N-AGATCGGAAGAG-CGTCGTGT-3′SpC3) was ligated by T4 RNA ligase 1 (NEB) by incubating at 25°C for 16 hours, and cDNA was recovered with the elution buffer of 95% (v/v) formamide and 10 mM EDTA (pH 8.0) by boiling at 95°C for 10 min, followed by ethanol precipitation. The library can be obtained by PCR amplification with next-generation sequencing primer and gel purification of size between 180 and 400 bp. Sequencing was performed at the University of Chicago Genomics Facility on an Illumina NovaSeq 6000 platform in single-end mode with 100 bp.

### Sequencing data analysis

#### Poly (A)^+^ RNA-seq and nuclear RNA-seq

Raw RNA-seq reads were trimmed with Cutadapt (version 4.6) (*68*). The reads were first aligned to the human rRNA using STAR (version 2.7.10a) (*69*) to further remove the rRNA contamination. The remaining unmapped reads were mapped to the human genome (GRCh38) with GENCODE v39 gene annotation using STAR (version 2.7.10a). Reads were assigned to gene regions using featureCounts (version 2.0.1) (*70*). nTPM (normalized transcripts per million) was calculated by RSEM (version 1.2.28) (*71*), and fold changes between different conditions were calculated by DESeq2 (version 1.38.3) (*72*). Intron retention events were assessed using IRFinder (version 1.3.0) (*49*) with default settings.

#### Reverse transcription–based RNA binding protein binding sites sequencing

FastQC (version 0.11.9) was used to assess the raw single-end FASTQ files. Cutadapt (version 4.3) was used for adapter trimming. Reads were first mapped to the human rRNA using STAR (version 2.7.10a) to further remove rRNA contamination. The remaining unmapped reads were aligned to the human reference genome (GRCh38) using STAR (version 2.7.10a). Only alignments with at least 24 matched bases were included for downstream analysis. Mapped reads were deduplicated using UMI-tools (version 1.1.1) and counted with featureCounts (version 2.0.1) (*70*). For gene-level *I*_NSE_ calculation, total reads per gene were calculated by the sum of reads mapped to introns and exons for each RNA-seq library. Bioconductor package DEseq2 (version 1.38.3) was then used to perform differential analysis between *N*_SON_ (or *N*_SRRM2_) and *N*_-priAB_ to calculate *I*_NSE_ (*72*). For *I*_NSE(intron)_ and *I*_NSE(exon)_ analysis, mapped reads were first assigned to intron and exon regions based on “Ensembl_canonical” exons. Regions between two successive canonical exons were defined as canonical introns. DEseq2 was again used to calculate *I*_NSE(intron)_ and *I*_NSE(exon)_. In the alternative analysis method using normalization to *N*_nu-RNA_, *N*_SON_ (or *N*_SRRM2_) was first subtracted by the sequencing depth-corrected *N*_-priAB_. That is, *N*_SON_corrected_ =*N*_SON_ − *F*_c_·*N*_-priAB_, where *F*_c_ is a correction factor given by the ratio of total mapped reads of ARTR-seq using SON antibody to the total mapped reads of ARTR-seq without primary antibody. *N*_SRRM2_corrected_ was defined similarly. DEseq2 analysis was then performed between *N*_SON_corrected_ (or *N*_SRRM2_corrected_) and *N*_nu-RNA_.

#### GO analysis

The functional enrichment analysis was performed using g:Profiler (version e110_eg57_p18_4b54a898) with g:SCS multiple testing correction method applying significance threshold of 0.05 and using Gene Ontology release 2023-07-27 (*73*).

#### Regression model

To identify the association of sequence features with enrichment, we used the regression coefficients of a GAM, as computed using the pyGAM library (*74*). The regression is given by the equation

$$\beta_{0}+f_{\text{GC}}\left(x_{\text{GC}}\right)+f_{\text{mil}}\left(x_{\text{mil}}\right)+\sum_{e}\left(\gamma_{\text{len}\left(e\right)}+\delta_{\text{category}\left(e\right)}\right)$$

where β_0_ is a constant bias term, *f*_GC_ is a learned spline function applied to the gene’s GC content (*x*_GC_), *f*_mil_ is a learned spline function applied to the base-2 logarithm of the gene’s mean intron length (*x*_mil_), the sum runs over all internal exons *e*, γ_1_, …, γ_7_ are scalar coefficients used to score the binned exon length [len(*e*)], and δ_1_, …, δ_81_ are scalar coefficients used to score the exon category [category(*e*)]. The exon category is obtained by quantile binning and combining four values: MaxEnt 3′ splice site score, MaxEnt 5′ splice site score (*44*), exon sequence ML score, and ±100-nt flanking intronic sequence ML score (specifically, upstream from −120 to −21 and downstream from +6 to +105) (*45*). Because the ML model was trained on exons of fixed length, it is unable to account for differences in exon lengths properly; therefore, instead of using the raw score, we used the linear regression residual of the score with respect to exon length. Our intron-centric analysis was performed similarly, the only difference being the use of an intron category instead of the exon category. Intron category is computed similarly to exon category, the only exception being the use of the ML score of the ±100-nt flanking exonic sequences instead of the exon sequence.

### Fluorescence labeling of FISH probe

DNA oligonucleotides were purchased from IDT. To prepare fluorescence-labeled probes, the 3′-end of oligonucleotides were first labeled with amine group as previously described (*75*). Briefly, to conjugate an amino–dideoxyuridine triphosphate (ddUTP) at the 3′ end of each oligonucleotide, 66.7 μM DNA oligonucleotides, 200 μM amino-11-ddUTP (Lumiprobe), and terminal deoxynucleotidyl transferase (0.4 U/μl, NEB) were mixed in 1× Terminal Transferase Reaction buffer and incubated at 37°C overnight. The reaction was cleaned up by P-6 Micro Bio-Spin Column (Bio-Rad). For fluorophore labeling, amine-modified DNA oligonucleotides were mixed with 25 μg of AF647 (Thermo Fisher Scientific) or CF568 (Sigma-Aldrich)–conjugated succinimidyl ester in 0.1 M sodium bicarbonate (pH 8.5) and incubated at 37°C overnight. The probes were cleaned up by ethanol precipitation and P-6 Micro Bio-Spin Columns. The labeling efficiency was calculated using NanoDrop One Spectrophotometer (Thermo Fisher Scientific no. ND-ONE-W). The average probe labeling efficiency was ~90%. The detailed sequences are provided in table S2.

### Fluorescence labeling of antibodies

The secondary antibodies against mouse (Jackson ImmunoResearch, no. 715-055-150, RRID:AB_2340777) and rabbit (Jackson ImmunoResearch, no. 711-005-152, RRID:AB_2340585) were labeled with AF488, CF568, or AF647 succinimidyl ester (Thermo Fisher Scientific). In brief, 24 μl of antibodies (1 mg/ml) was mixed with 3 μl of 10× phosphate-buffered saline (PBS) and 3 μl of sodium bicarbonate (1 M, pH 8.5), and 1 μl of dimethyl sulfoxide dissolved fluorophore (1 μg/μl) was added to the reaction and incubated at room temperature for 1 hour. The labeled antibodies are purified by P-6 Micro Bio-Spin Columns (Bio-Rad).

### RNA fluorescence in situ hybridization

RNA FISH probes were designed using a Stellaris probe designer and labeled as described above. After removing the medium and washing once with 1× PBS, the cells were fixed with 4% PFA (Electron Microscopy Sciences) in 1× PBS at room temperature for 10 min. Cells were washed three times with 1× PBS and permeabilized with a solution containing 0.5% Triton X-100 (Thermo Fisher Scientific) and 2 mM vanadyl ribonucleoside complexes (Sigma-Aldrich, no. R3380) in 1× PBS on ice. Cells were washed three times with 1× PBS, once with 2× saline-sodium citrate buffer (SSC), and once with wash buffer [10% formamide (Ambion, no. AM9342) in 2× SSC]. Cells were then incubated with FISH probes in hybridization buffer [10% formamide, 10% (w/v) dextran sulfate, and 10 mM dithiothreitol (DTT) in 2× SSC] at a final concentration of 1 nM per probe for 16 hours at 37°C in the dark. After hybridization, cells were washed twice with wash buffer for 15 min at 37°C before being used for following immunostaining or imaging.

### DNA fluorescence in situ hybridization

#### Cas9 targeting site and probe design

The Cas9 binding site against *LAMA5* genomic region was designed using CRISPR Guide RNA Design Tool using Benchling. To avoid interference with RNA FISH, the antisense strand was used for designing guide RNA and DNA FISH probes. The average spacing between each Cas9 binding site is 300 bp. Template DNAs with T7 promoter region for generating crRNAs (CRISPR RNA) were purchased from IDT. We designed DNA FISH probes by loading sequences between adjacent Cas9 binding sites into Oligoarray 2.1 (*76*) with the following conditions: length, 18 to 30 nt; melting temperature (*T*m), 72° to 90°C; GC content, 30 to 70%; *T*m threshold for secondary structure formation, 54°C; minimal *T*m to consider cross-hybridization, 54°C; prohibited sequences, GGGG; CCCC; TTTTT; AAAAA; the minimum distance between the 5′ ends of two adjacent oligonucleotides, 30; the maximum number of oligonucleotides to design per input sequence, 30; maximum distance between the 5′ end of the oligonucleotide and the 3′ end of the input sequence, 1000. Probes with multiple BLAST alignments were then removed to avoid nonspecific binding. Designed probes were purchased from IDT and labeled with AF647 using the same protocol as shown in the “Fluorescence labeling of FISH probe” section.

#### Preparation of guide RNAs

crRNAs were synthesized using HiScribe T7 Quick High Yield RNA Synthesis Kit (NEB) according to the manufacturer’s instructions. All crRNAs are transcribed together; to make the transcription efficiency the same for different crRNAs, we used a 10-nt common region to 5′-end of each crRNA to make the transfection efficiency homogeneous (*77*). The synthesized crRNAs were purified using polyacrylamide gel electrophoresis. The guide RNA was assembled using 1:1 ratio of purified crRNAs and the Alt-R CRISPR-Cas9 tracrRNA from IDT in Nuclease-Free Duplex Buffer (IDT), incubated at 95°C for 5 min, and slowly cooled down to room temperature over 1 hour.

#### GOLD FISH and RNA FISH

To simultaneously detect DNA and RNA, we adapted the previously published GOLD FISH protocol (*37*). Briefly, cells were first fixed using prechilled MAA solution (methanol and acetic acid mixed in 1:1 ratio) at −20°C for 20 min and washed three times with 1× PBS and once with blocking-binding buffer [BBB; 20 mM Hepes (pH 7.5), 100 mM KCl, 5 mM MgCl_2_, 5% (v/v) glycerol, 0.1% (v/v) Tween 20, 1% (w/v) BSA with fresh added 1 mM DTT, *Escherichia coli* tRNA (0.1 mg/ml), and RNaseOUT (1 U/μl)]. After fixation, Cas9_H840_ (a gift from the laboratory of T. Ha) was assembled with annealed guide RNA in 1:1.4 ratio for 10 min at room temperature in BBB buffer. The cells were then incubated with assembled Cas9 RNP for 1 hour at 37°C. After incubation, 300 nM Rep-X (a gift from the laboratory of T. Ha) in BBB buffer supplemented with 2 mM adenosine triphosphate was added to cells and incubated at 37°C for 45 min. The cells were washed three times with 1× PBS, once with 2× SSC, and once with 1× wash buffer. Cells were then incubated with DNA FISH probes (1 nM per probe) and RNA FISH probes (1 nM per probe) in hybridization buffer supplemented with RNaseOUT (1 U/μl) for 4 hours at 37°C in the dark. After hybridization, cells were washed twice with wash buffer at 37°C for 15 min.

### Immunofluorescence staining

After DNA and RNA FISH or RNA FISH alone, cells were fixed again with 4% PFA in 1× PBS for 10 min, washed three times with 1× PBS, and blocked with UltraPure BSA (1 mg/ml) (50 mg/ml, Invitrogen) in 1× PBS for 30 min. Cells were immunostained with rabbit anti-SON antibody (1:200 dilution, Novus, RRID:AB_11006030), mouse anti-SRRM2 antibody (1:200 dilution, Sigma-Aldrich, RRID:AB_477511), or mouse anti-SRSF1 antibody (1:250 dilution, Invitrogen, RRID:AB_2533080) for 1 hour at room temperature followed by three-times wash with 1× PBS. Cells were then incubated with labeled secondary antibody (1200 dilution) for 1 hour at room temperature and washed three times again with 1× PBS.

### Fluorescence imaging

Before imaging, nuclei were stained with 4′,6-diamidino-2-phenylindole (DAPI) for 10 min and washed once with 1× PBS before imaging. To reduce photobleaching, 100 μl of imaging buffer containing tris-HCl (50 mM, pH = 8), 10% glucose, catalase (67 μg/ml, Sigma-Aldrich), and glucose oxidase (0.5 mg/ml, Sigma-Aldrich) in 2× SSC was used before imaging. For RNA FISH with immunostaining, imaging was performed on a Nikon Ti2-E inverted confocal microscope (Nikon AX-R) using either a CFI (Chromatic aberration-Free Infinity) Plan Apo objective [60× oil, numerical aperture (NA) 1.40, Nikon] and GaAsP photomultiplier tube (PMT) detectors (DUX-ST detectors, Nikon). The pinhole size was maintained at 2 AU. Sample excitation was performed using the AS405/488/561/640 laser unit (LUA-S4, Nikon) with appropriate laser and filter settings. Z-stacks (0.2-μm step size, seven stacks) were taken for each channel, and an artificial intelligence–based denoising (Nikon NIS-Elements AR 5.41.02 software) was applied. For GOLD FISH and RNA FISH with immunostaining, imaging was performed using a Nikon TiE microscope with a CFI HP objective (100×, NA 1.49, Nikon), and an electron-multiplying charge-coupled device (EMCCD) (Andor, iXon Ultra 888). Samples were excited with a 647-nm laser (Cobolt MLD), a 561-nm laser (Coherent Obis), a 488-nm laser (Cobolt MLD), and a 405-nm laser (CL2000, Crystal Laser).

### Image analysis

Nikon NIS-Elements software (AR 5.41.02), Fiji ImageJ2, and MATLAB R2022b were used for image analysis.

#### Quantification of R_NS/cell_ and R_NS/NU_

Images were first denoised by NIS-Elements. Fiji was then used for maximum intensity projection and channel splitting. For cell segmentation, we used a Cellpose cyto2 model on the RNA channel (*78*, *79*). The denoised images and generated masks were subsequently analyzed in MATLAB with customized codes. The nuclei were segmented in the DAPI channel by Otsu’s algorithm, and nuclear speckles were segmented in the SON or SRRM2 channel based on a global intensity threshold. Single-cell *R*_NS/cell_ or *R*_NS/NU_ values were then calculated by determining the mean RNA fluorescence intensity in nuclear speckles and dividing it by the cellular or nucleoplasm mean intensity.

#### Quantification of DNA and RNA foci

In epifluorescence images of GOLD FISH, RNA FISH with speckle immunostaining, individual nucleus was manually selected in Fiji and saved as.tif files, followed by automated analysis in MATLAB with customized codes. For the DNA channel, a difference of Gaussians (DoG) filter was first used for background subtraction. Subsequently, DNA foci were identified by applying a global intensity threshold based on the mean and SD of image intensity after the DoG transformation. A size threshold of 12 pixels was applied using MATLAB’s built-in function bwareaopen, and the regionprops function was used to extract centroid and area of each focus. RNA foci and nuclear speckles were identified using a similar approach, with a size threshold of 15 pixels. For each DNA or RNA focus, we calculated its center-to-center distance to the nearest nuclear speckle. This distance was then normalized by the sum of the radii of nuclear speckle and the RNA/DNA focus. An RNA or DNA focus was deemed nuclear speckle associated if the normalized center-to-center distance was less than 1.4. In the confocal images of RNA FISH with speckle immunostaining, individual RNA focus was identified with the same procedure. An RNA focus was deemed nuclear speckle associated if the normalized center-to-center distance was less than 1.2.

### Reverse transcription polymerase chain reaction

Total RNA was extracted using TRIzol Reagent (Invitrogen) according to the manufacturer’s instructions. Residual DNA contamination in extracted RNA was removed using Turbo DNase (Invitrogen). RNA (1 μg) was reverse transcribed using iScript cDNA Synthesis Kit (Bio-Rad), and PCR was performed using Q5 High-Fidelity 2X Master Mix (NEB). The fraction of unexcised intron is monitored using RT-PCR with primers located on two adjacent exons. The primer specificity is checked using Primer Blast. All primers are listed in table S2. Amplified products were separated on a 1.5% agarose/tris-acetate-EDTA gel with ethidium bromide staining and visualized on a Bio-Rad ChemiDoc Imager. The bands were quantified by Fiji.

### Cell viability assay

Cell viability was assessed by measuring dead cells ratio stained with trypan blue using a fluorescence microscope (*80*). Cells were seeded in an eight-well imaging chamber; after SON and SRRM2 double knockdown or treatment with control siRNA, the cells were stressed at 43°C for 2 hours. After heat shock, the cells were washed once with 1× PBS and stained sequentially with 1:10 dilution of Trypan Blue Solution (0.4%, Thermo Fisher Scientific) for 3 min and 1:500 of Hoechst 33342 (20 mM, Thermo Fisher Scientific) for 10 min. Imaging was performed on a Nikon Ti2-E inverted confocal microscope (Nikon AX-R) using a Plan Fluor objective (20× air, NA 0.50, Nikon) and GaAsP PMT detectors (DUX-ST detectors, Nikon). Cell viability was measured using Stardist as a plugin in Fiji (*81*).
