# Supplementary material for: Dynamics of RNA localization to nuclear speckles are connected to splicing efficiency
Source: Sci Adv. 2024 Oct 16;10(42):eadp7727. doi: 10.1126/sciadv.adp7727 (PMC11482332; doi:10.1126/sciadv.adp7727)
Supplement: Supplementary file 1 — Figs. S1 to S15 Legends for tables S1 to S6 [file sciadv.adp7727_sm.pdf]

Science Advances
AAAS

Supplementary Materials for

Dynamics of RNA localization to nuclear speckles are connected to splicing efficiency

Jinjun Wu *et al.*

Corresponding author: Jingyi Fei, jingyifei@uchicago.edu; Oded Regev, regev@cims.nyu.edu

Sci. Adv. **10**, eadp7727 (2024)
DOI: 10.1126/sciadv.adp7727

The PDF file includes:

Figs. S1 to S15
Legends for tables S1 to S6
References

Other Supplementary Material for this manuscript includes the following:

Tables S1 to S6

Supplementary figures

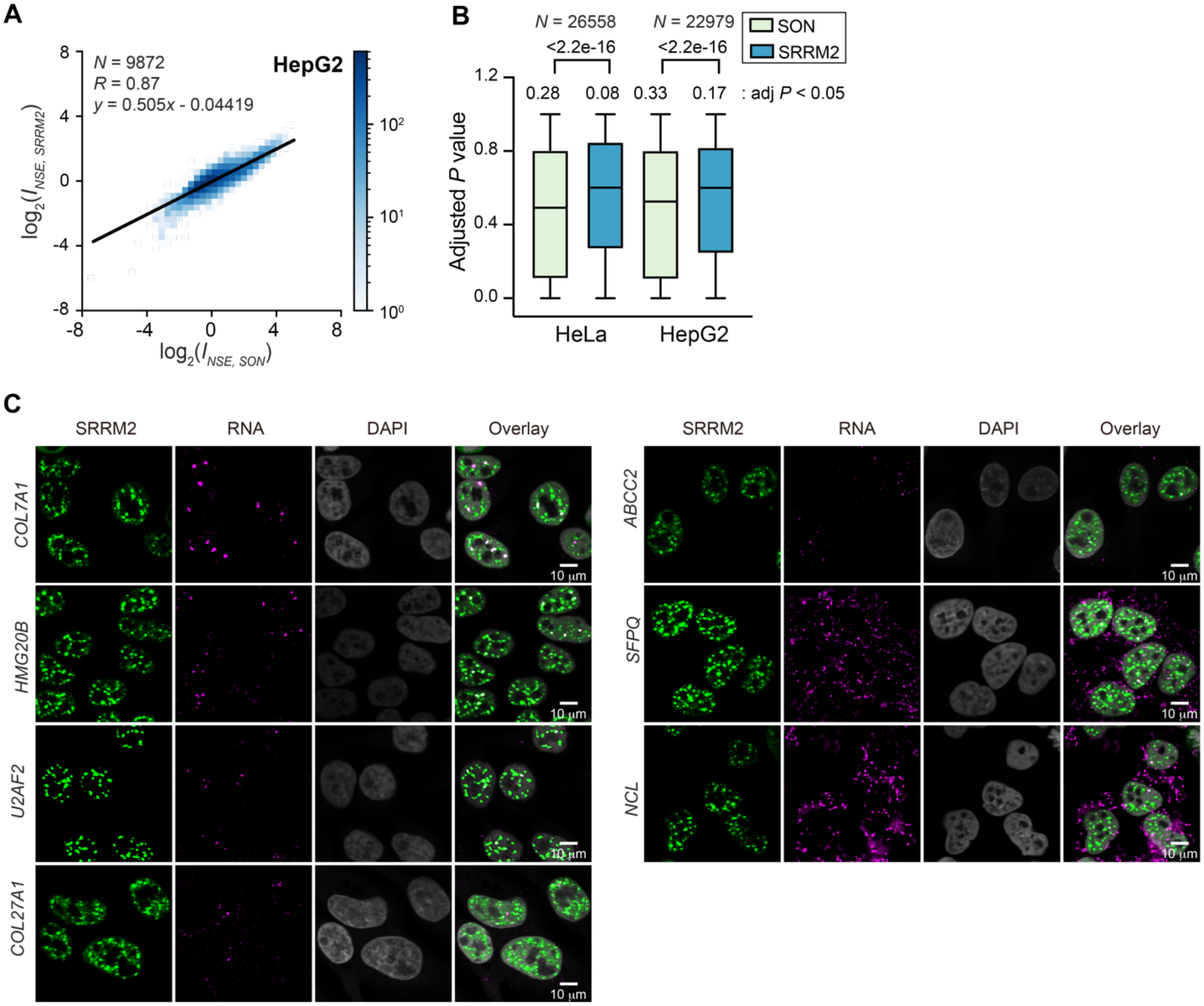

Figure S1. Additional analysis and validation of I_{NSE} calculation. (A) 2D histogram showing the correlation between I_{NSE} determined through targeting SON and SRRM2 in HepG2 cells. Genes with *lfcSE*<1 from DESeq analysis of ARTR-seq are included. "*N*" reports the total number of genes included in each plot, and "*R*" reports Pearson's correlation coefficient. Linear function ($y = ax+b$) is used to fit $I_{NSE,SRRM2}$ to $I_{NSE,SON}$ and labeled in the 2D histogram. (B) Comparison of the adjusted *P*-value for each gene in DESeq analysis of speckle enrichment between targeting SON and SRRM2 in HeLa and HepG2 cells. Data using SON antibody shows less variation than data using SRRM2 antibody. *P*-values are calculated using unpaired t-test. "*N*" reports the number of genes included in each comparison. The fraction of genes with adjusted *P*-value less than 0.05 (adj $P < 0.05$) in each case is labeled above each box plot. (C) Additional RNA FISH imaging validation of ARTR-seq on selected genes. *COL7A1*, *HMG20B*, *U2AF2, SFPQ* and *NCL* transcripts were imaged in HeLa cells. *COL27A1* and *ABCC2* transcripts were imaged in HepG2 cells. Nuclear speckles were stained with AF488-labeled antibodies against SRRM2 (green), RNAs were labeled with AF647-labeled FISH probes (magenta), and nuclei were stained with DAPI (grey). Scale bar: 10 µm.

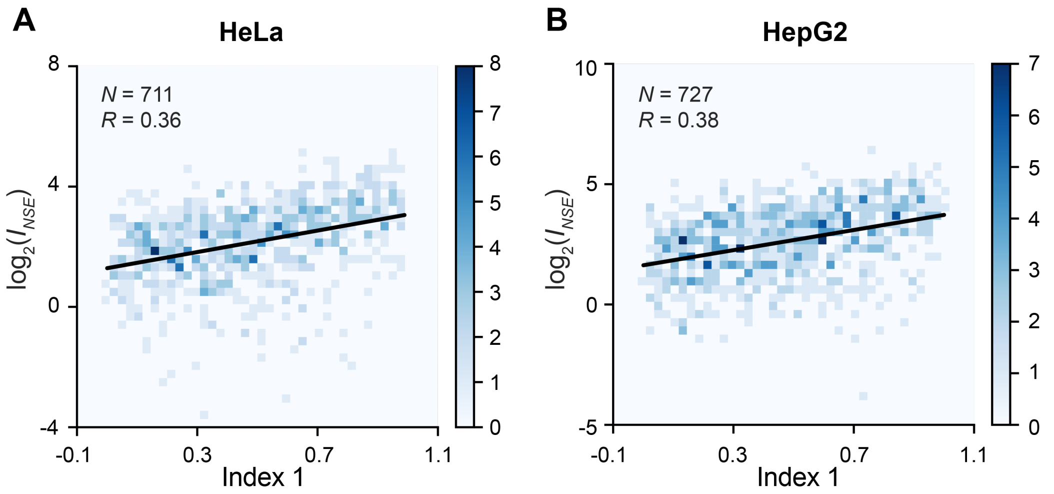

Figure S2: Comparison between ARTR-seq and APEX-seq methods for nuclear speckle transcriptome analysis. 2D histogram showing the correlation between I_{NSE} (in $\log_2$ scale) determined by ARTR-seq in HeLa cells (A) or HepG2 cells (B) and Index 1 from APEX-seq (*5*). A higher Index 1 indicates higher speckle enrichment. Genes with *lfcSE*<1 from DESeq analysis of ARTR-seq, and with Index 2 > 0.5 in APEX-seq are included. This Index 2 threshold filters out genes exhibiting large expression level change upon expressing APEX-fused proteins. "*N*" reports the total number of genes included in each plot, and "*R*" reports Pearson's correlation coefficient.

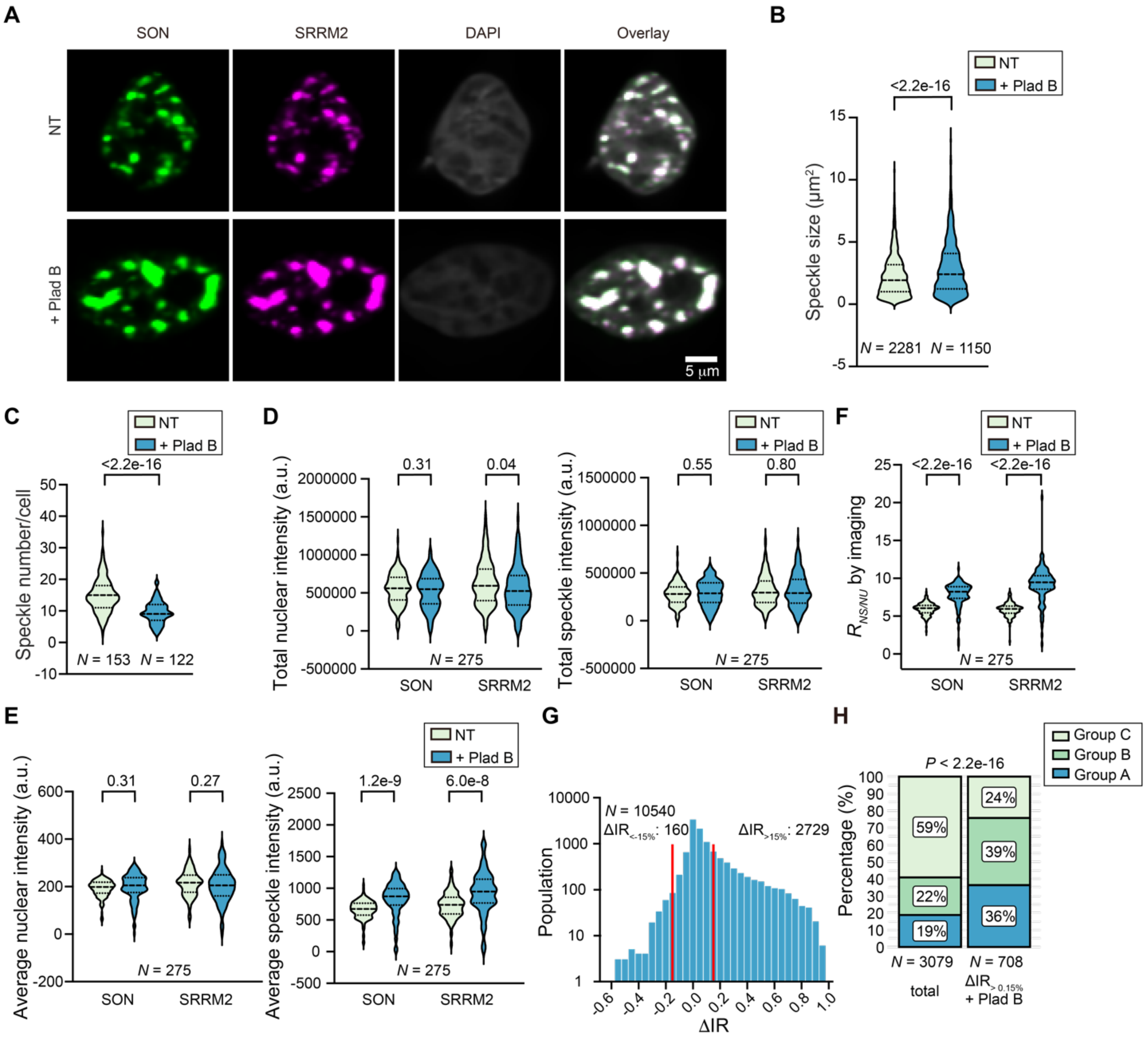

Figure S3: Impact of Plad B treatment on nuclear speckles. (A) Representative images of co-staining of SON and SRRM2 in nuclear speckles in the no treatment (NT) and Plad B treatment (+Plad B) conditions. SON was stained with AF488 labeled antibodies (green). SRRM2 was stained with CF568 labeled antibodies (magenta). Nuclei were stained with DAPI (grey). Scale bar: 5 µm. Violin plots of (B) speckle size, (C) number of speckles per cell, (D) total SON and SRRM2 intensity in the nucleus of each cell (left) and in speckles of each cell (right), (E) average SON and SRRM2 intensity in the nucleus of each cell (left) and in speckles of each cell (right). (F) Nuclear speckle enrichment of SON and SRRM2 relative to nucleoplasm ($R_{NS/NU}$) is calculated as the ratio of the average intensity of SON/SRRM2 signal in speckles to the average intensity in the nucleoplasm. (G) IRFinder analysis (*49*) to identify intron excision affected by Plad B treatment. The number of intron retention events with more than 15% increase ($\Delta IR_{>15\%}$) or decrease ($\Delta IR_{<-15\%}$) are labeled. (H) Percentage of Group A, B and C genes without and with taking the subset of genes containing $\Delta IR_{>15\%}$ introns. "*N*" reports total number of speckles in (B), total number of cells in (C)-(F), number of introns identified in (G) and number of genes in (H). *P*-values are calculated using unpaired t-test in (B)-(F) and using Fisher's exact test in (H).

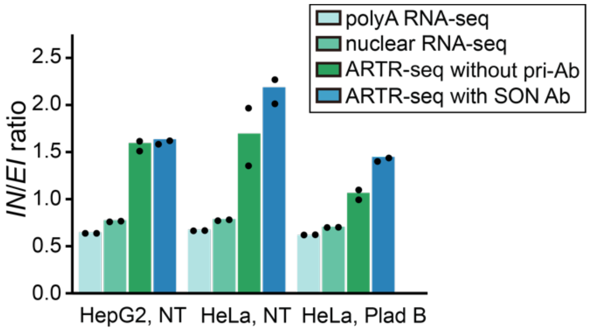

Figure S4: The ratio between the number of reads mapped to an intronic position 100 nt from a splice site (*IN*) to those mapped to an exon-intron boundary (*EI*). Without any intron lariats or lariat intermediates, a theoretical value of 1 would be expected, since each unexcised intron has the same contribution to the numerator and to the denominator. Intron lariats or lariat intermediates are expected to increase this value because they contribute more to the numerator than to the denominator. Consistent with the rapid degradation of lariats (*32*), nuclear RNA-seq and polyA RNA-seq do not show values greater than 1, suggesting insignificant impact of lariats. The discrepancy from the theoretical value is likely attributable to sequencing biases. We therefore do not expect ARTR-seq to capture a significant number of lariats or lariat intermediates. Indeed, both ARTR-seq with SON antibody and without a primary antibody show a similar ratio larger than 1, also likely due to sequencing biases in this method. Specifically, it is possible that in situ reverse transcription in crosslinked samples may be sensitive to the presence of spliceosomal complexes around splice sites, slightly reducing the number of *EI* reads.

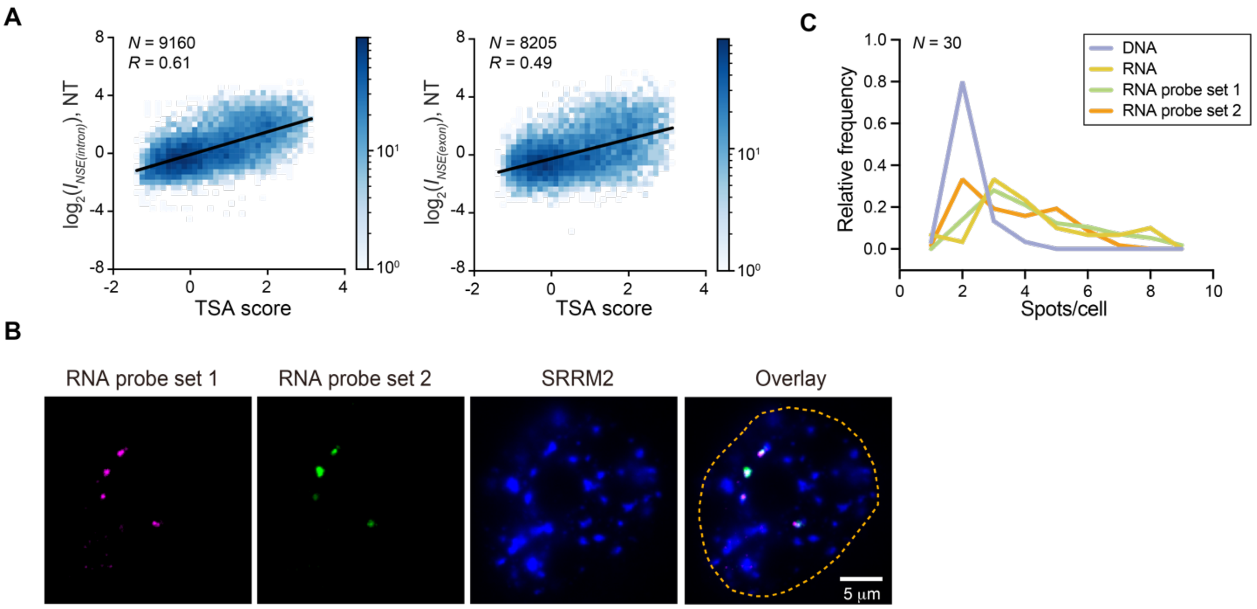

Figure S5: Comparison between ARTR-seq and TSA-seq in HepG2 cells and additional control images for GOLD FISH and RNA FISH. (A) 2D histogram showing the correlation between $I_{NSE(intron)}$ or $I_{NSE(exon)}$ (in $\log_2$ scale) determined by ARTR-seq in HepG2 cells and TSA score from TSA-seq in K562 cells. Genes with higher TSA scores are closer to speckles. (B) To exclude the possibility that the combination of GOLD FISH and RNA FISH compromised the specificity of RNA FISH, intron-targeting RNA FISH probes for *LAMA5* transcripts are split into two sets labeled with AF647 and CF568 (RNA probe set 1 and RNA probe set 2). Specific RNA probe binding should generate colocalized signals from the two RNA probe sets. The two-color RNA FISH signals show ~86% colocalization, validating the specificity of transcript detection. (C) Distributions of the number of RNA foci detected using RNA probe set 1 and RNA probe set 2 are consistent with the number of RNA foci detected in the GOLD FISH and RNA FISH co-staining (data from Figure 4I), further validating that the combination of GOLD FISH and RNA FISH did not compromise the accuracy of RNA foci detection. "*N*" reports total number of cells included in the histogram, collected from 3 biological replicates.

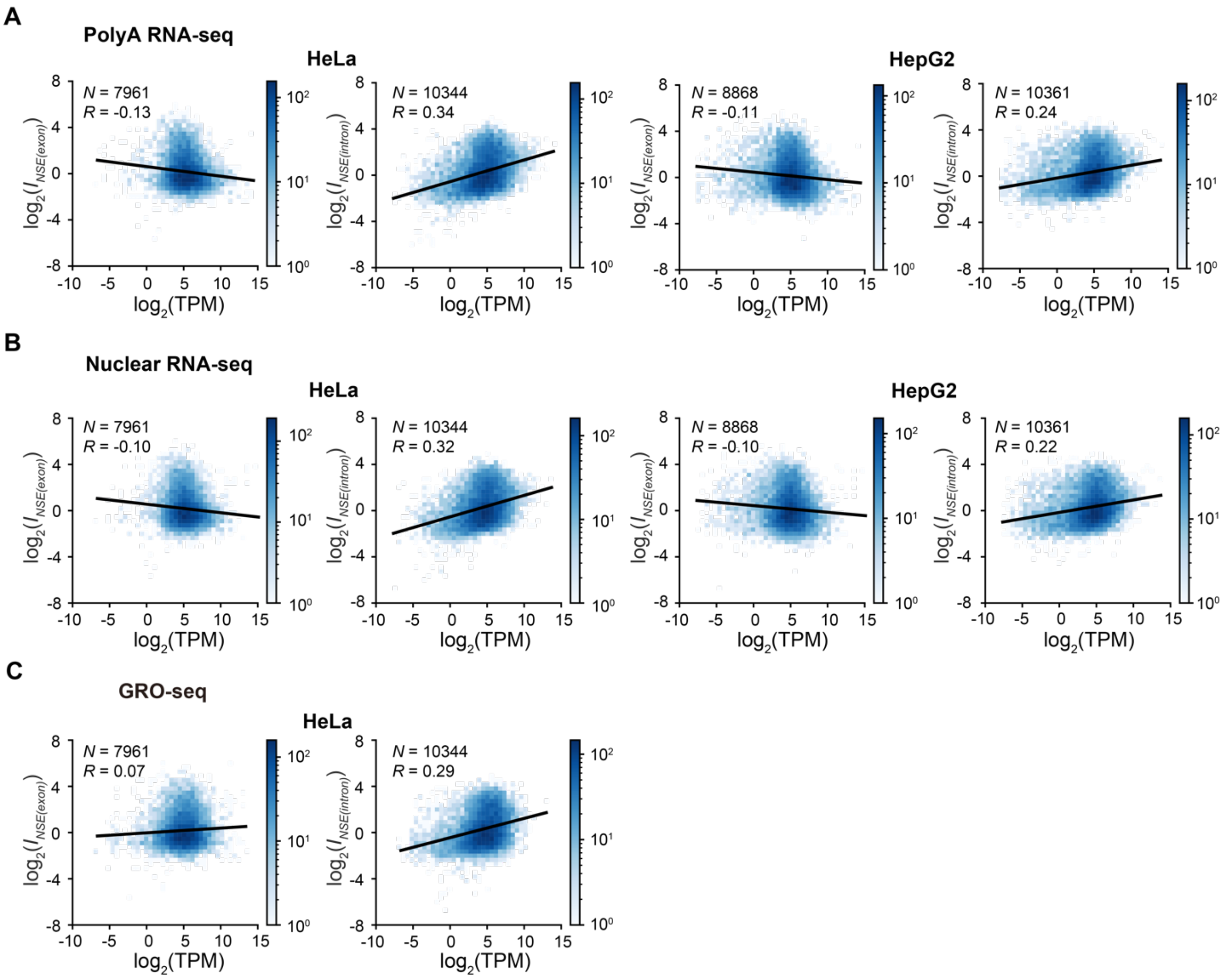

Figure S6: Correlation between transcript speckle enrichment and transcript abundance. 2D histogram showing the correlation between $I_{NSE(exon)}$ or $I_{NSE(intron)}$ determined by ARTR-seq using TPM (in $\log_2$ scale) from (A) polyA RNA-seq in HeLa cells or HepG2 cells; (B) rRNA-depleted nuclear RNA-seq in Hela cells or HepG2 cells; (C) GRO-seq in HeLa cells (*38*). TPM: transcripts per million reads. TPM was calculated by RSEM (*71*). Genes with *lfcSE*<1 from DESeq analysis of ARTR-seq are included. "*N*" reports number of genes included in each plot, and "*R*" reports Pearson's correlation coefficient.

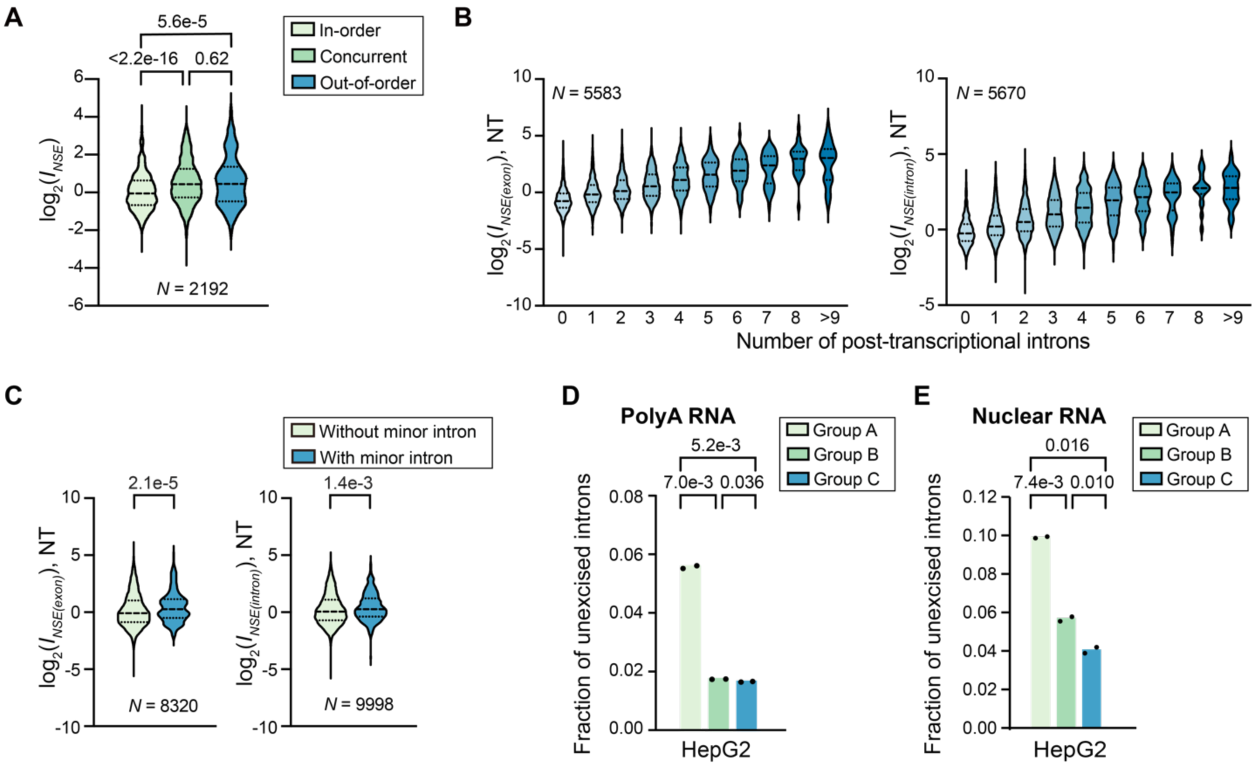

Figure S7: Transcript speckle enrichment is associated with splicing timing and efficiency in HepG2 cells. (A) Violin plot comparing I_{NSE} values of in-order excised introns, concurrently excised introns, and out-of-order excised introns, as classified by CoLa-seq (*39*). (B) Violin plot comparing $I_{NSE(exon)}$ or $I_{NSE(intron)}$ values of transcripts containing different numbers of post-transcriptionally excised introns, as characterized by nanopore RNA-seq (*40*). (C) Violin plot comparing $I_{NSE(exon)}$ or $I_{NSE(intron)}$ values of transcripts containing minor splice sites and those without (*42*). In (A) and (C), *P*-values calculated with unpaired t-tests are reported above each violin plot. "*N*" reports the total number of introns or genes in each comparison. (D-E) Fraction of unexcised introns (*EI*/(*EI*+*EE*)), calculated as in Figure 2A, for Group A, B and C genes under NT conditions at the polyA (determined from polyA RNA-seq) and nuclear RNA level (determined by nuclear RNA-seq). The two RNA-seq replicates were calculated individually. Each bar in (D)-(E) reports the mean of the two replicates. *P*-values are calculated with unpaired t-tests.

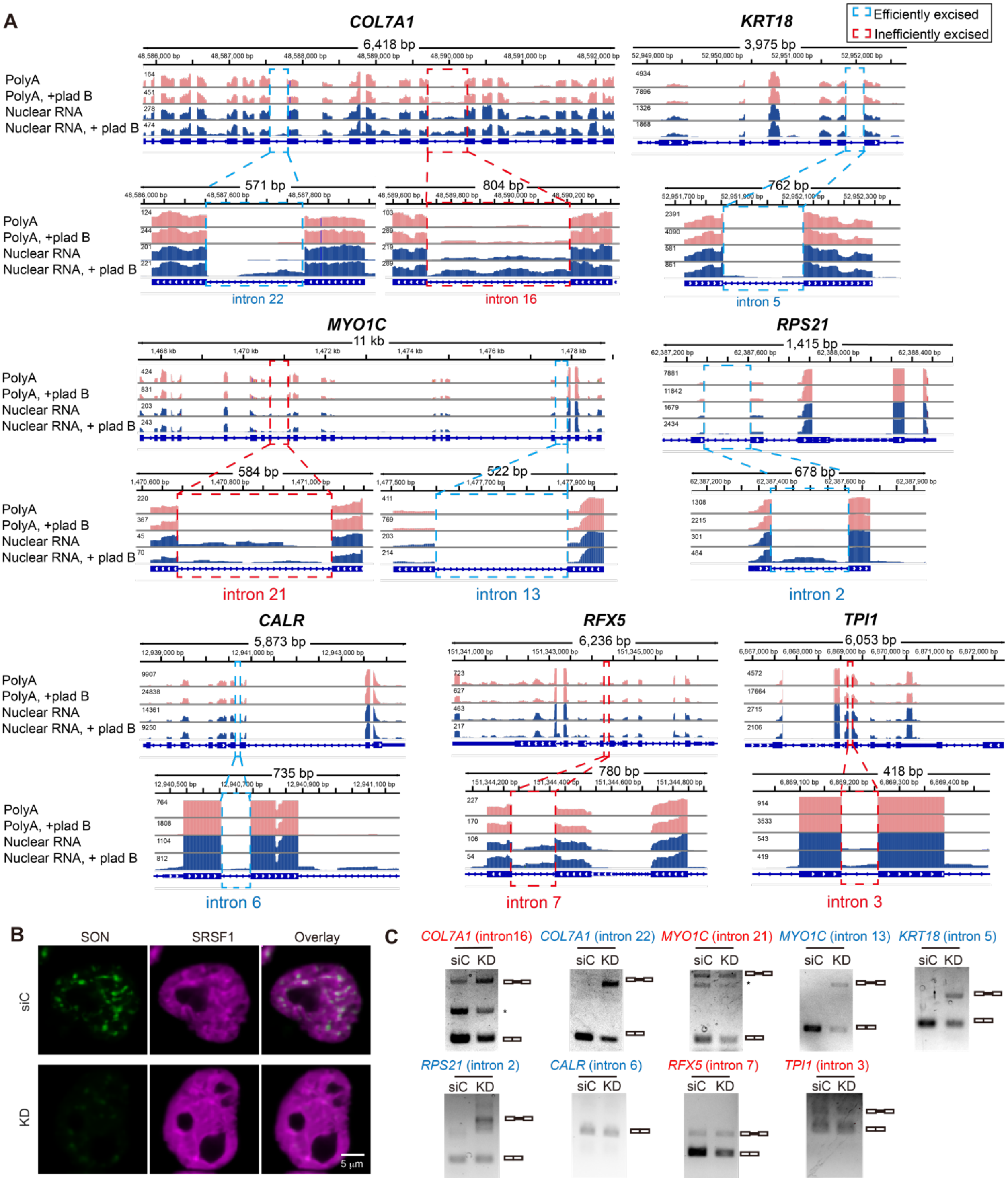

Figure S8: Genome tracks and electrophoresis analysis of additional selected genes in RT-PCR assay. (A) Genome tracks showing polyA RNA-seq (pink) and nuclear RNA-seq (blue) under NT and Plad B treatment conditions for additional selected Group A genes (*COL7A1*, *MYO1C*), Group B genes (*KRT18*, *RPS21*) and Group C genes (*CALR, RFX5, TPI1*). Selected introns are highlighted in cyan and red for efficiently excised introns and inefficiently excised introns respectively. (B) Representative images of immunofluorescence staining of SON (using

AF488, green) and SRSF1 (using CF568, magenta). SRSF1 is moderately enriched in nuclear speckles and is uniformly distributed throughout nucleoplasm upon speckle disruption. Scale bar: 5 µm. (C) Electrophoresis analysis of RT-PCR products from Group A gene (*COL7A1*, *MYO1C*), Group B gene (*KRT18*, *RPS21*), and Group C genes (*CALR, RFX5, TPI1*), either upon double knockdown of *SON* and *SRRM2* (KD), or treatment with control siRNA (siC). Asterisks denote unknown bands, which were not considered in the calculation of apparent fraction of unexcised intron.

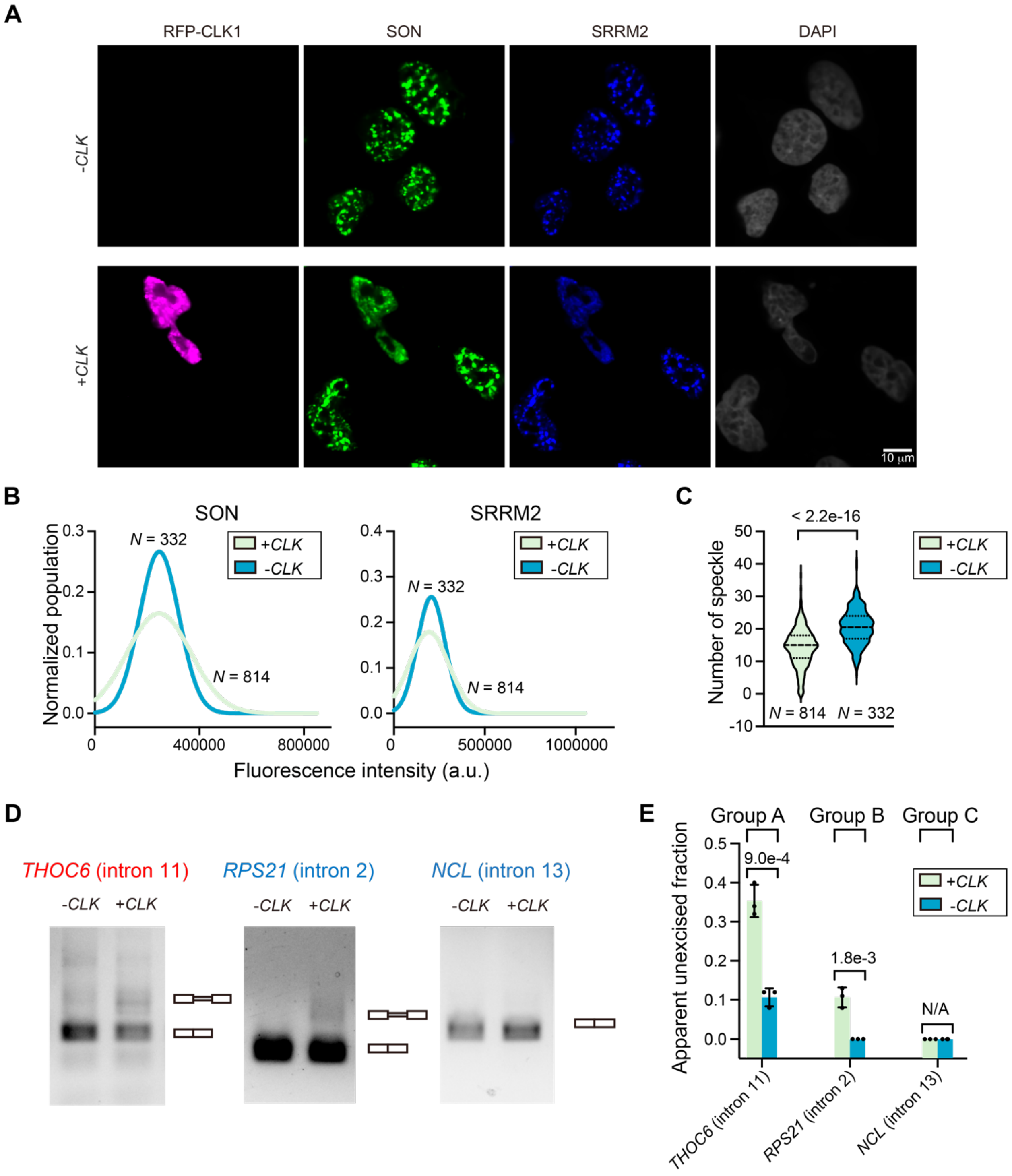

Figure S9: **Effect of speckle disruption by *CLK1* overexpression on splicing.** (A) Representative immunofluorescence images showing nuclear speckles in control (-*CLK*) and *CLK1* overexpressed (+*CLK*) cells. Nuclear speckles were stained with AF488 labeled antibody against SON antibody (AF488, green) and SRRM2 (AF647, blue). Nuclei were stained with DAPI (grey). Cells with *RFP-CLK1* overexpression show high RFP signal (magenta). Scale bar: 10 μm. (B) Histograms of SON and SRRM2 immunofluorescence intensity distribution of cells with and

without *CLK1* overexpression. (C) Violin plot showing the number of speckles per cell with and without *CLK1* overexpression. "*N*" reports total number of cells included in each plot in (B) and (C). Intensity threshold was applied based on the total intensity of SON and SRRM2 to select nuclear speckles. (D) Representative electrophoresis analysis of RT-PCR products from *THOC6* (intron 11), *RPS21* (intron 2) and *NCL* (intron 13) in control and *CLK1* overexpressed cells. (E) Apparent unexcised fractions of selected introns are calculated by ratios of the intensity of the unexcised band and the sum of the unexcised band and excised band. The intensity of bands was quantified using Fiji. Error bars report standard deviation from three biological replicates. *P*-values are calculated with unpaired t-tests in (C) and (E).

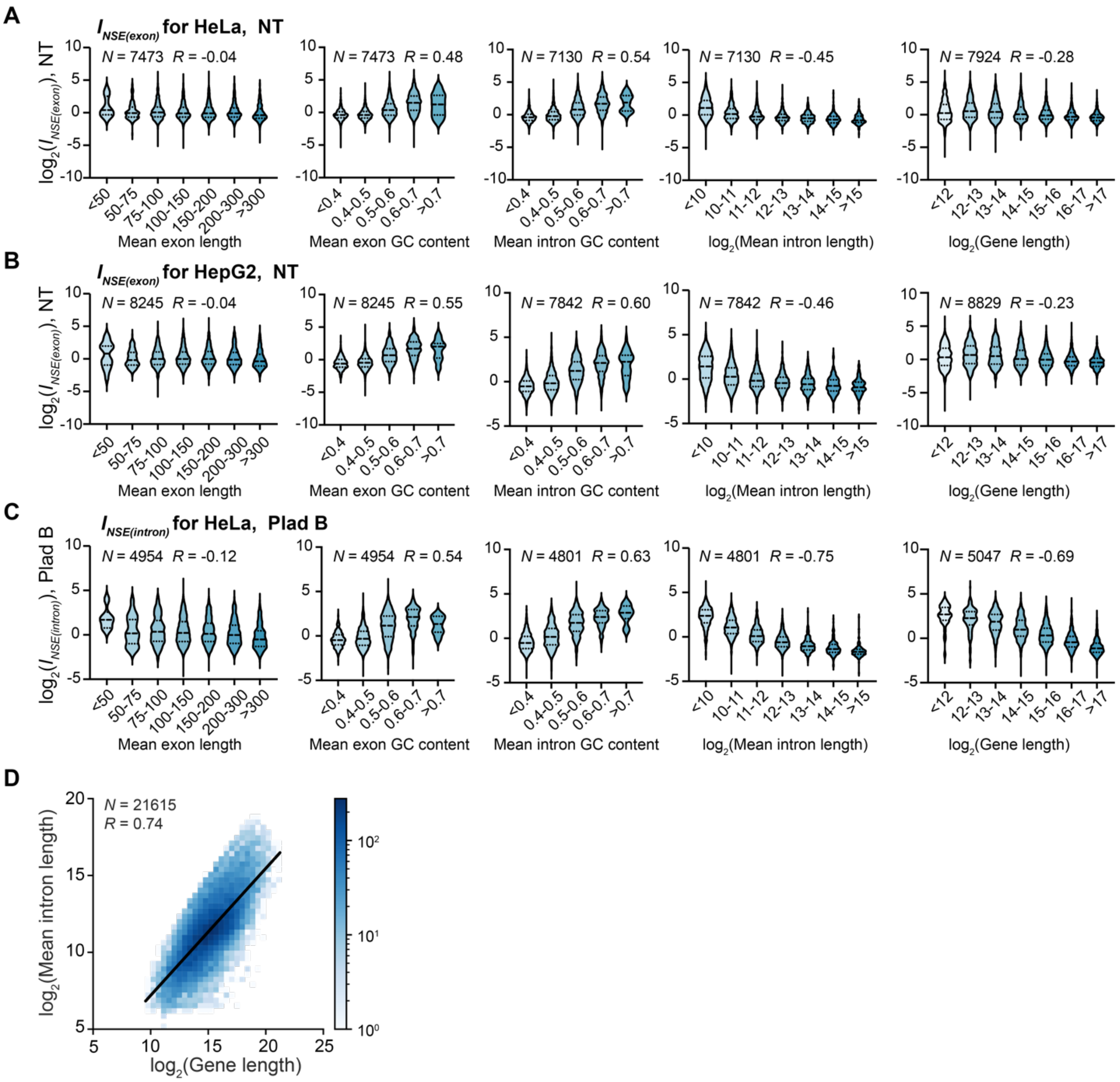

Figure S10: RNA sequence features correlated with speckle enrichment. Comparison of transcript speckle enrichment in each bin based on mean exon length, mean exonic GC content, mean intronic GC content, mean intron length ($\log_2$ scale) and gene length ($\log_2$ scale) of each transcript for (A) $I_{NSE(exon)}$ HeLa cells under NT condition; (B) $I_{NSE(exon)}$ in HepG2 cells under NT condition; (C) $I_{NSE(intron)}$ in HeLa cells with Plad B treatment. Genes with *lfcSE*<1 from DESeq analysis of ARTR-seq are included. "*N*" reports total number of genes included in each plot. "*R*" reports Pearson's correlation coefficient calculated using unbinned data in each plot. (D) 2D histogram showing the correlation between mean intron length and total gene length. "*N*" reports total number of genes. "*R*" reports Pearson's correlation coefficient.

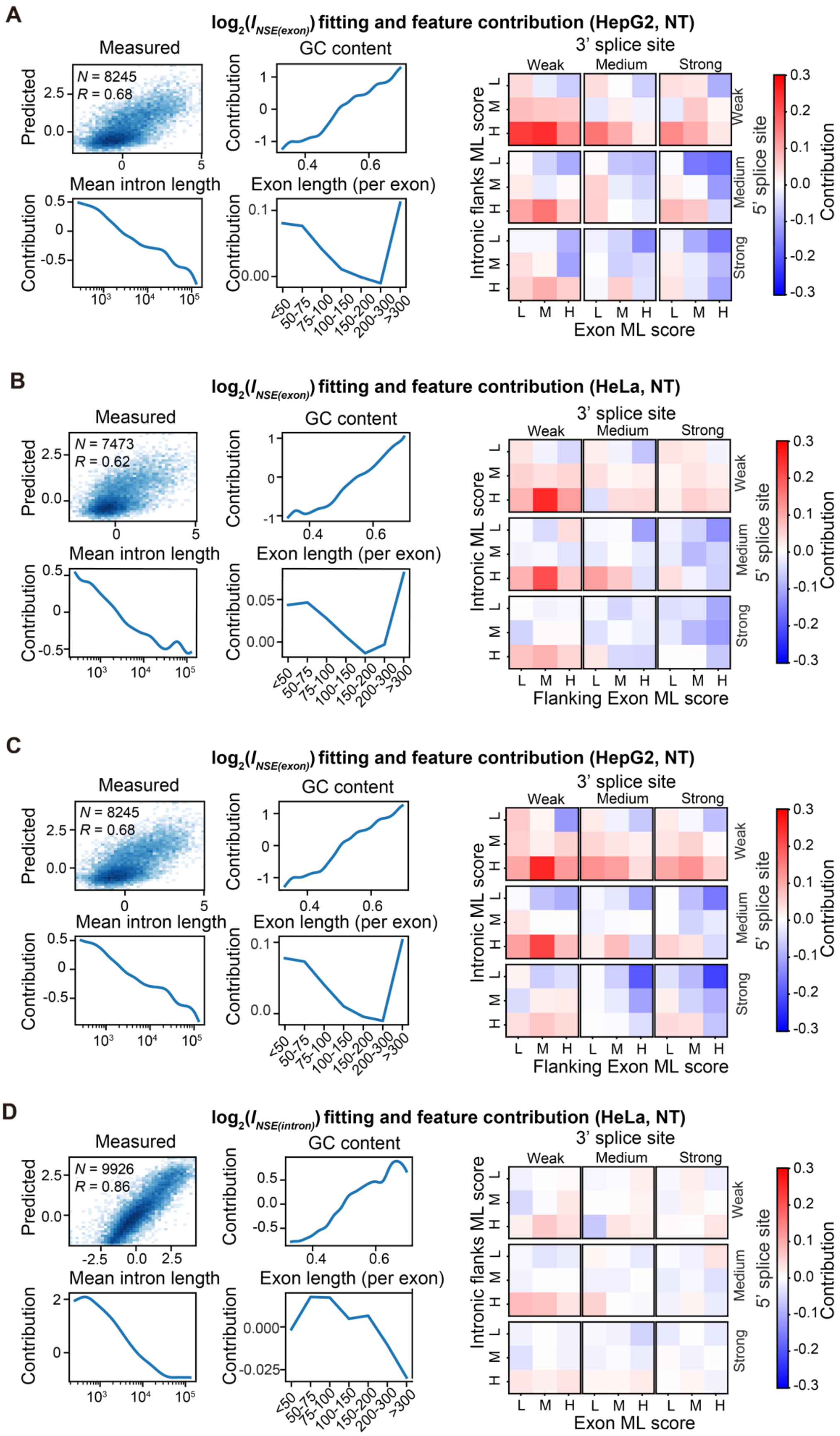

A
log2(I_NSE(exon)) fitting and feature contribution (HepG2, NT)
Measured
Predicted
N = 8245
R = 0.68
GC content
Contribution
Mean intron length
Exon length (per exon)
<50
50-75
75-100
100-150
150-200
200-300
>300
3' splice site
Weak
Medium
Strong
Intronic flanks ML score
5' splice site
Exon ML score
L
M
H
B
log2(I_NSE(exon)) fitting and feature contribution (HeLa, NT)
N = 7473
R = 0.62
Flanking Exon ML score
Intronic ML score
C
log2(I_NSE(exon)) fitting and feature contribution (HepG2, NT)
N = 8245
R = 0.68
Intronic ML score
Flanking Exon ML score
D
log2(I_NSE(intron)) fitting and feature contribution (HeLa, NT)
N = 9926
R = 0.86
Intronic flanks ML score
Exon ML score

Figure S11: Additional regression analyses on transcript speckle enrichment under NT condition. Input parameters and other related details of the regression model are described in the main text and Methods. (A) The exon-centric regression model reveals contributions from GC content, mean intron length, individual exon length, and a combination of splice site strength, exonic ML score and flanking intronic ML score to the transcript speckle enrichment $I_{NSE(exon)}$ values under NT in HepG2 cells. (B-C) The intron-centric regression model reveals contributions from GC content, mean intron length, individual exon length, and a combination of splice site strength, intronic ML score and flanking exonic ML score to $I_{NSE(exon)}$ values in HeLa cells under NT (B), and in HepG2 cells under NT (C). (D) The exon-centric regression analysis on $I_{NSE(intron)}$ values in HeLa cells under NT. "*N*" reports the number of genes in the regression analysis, and "*R*" reports Pearson's correlation coefficient.

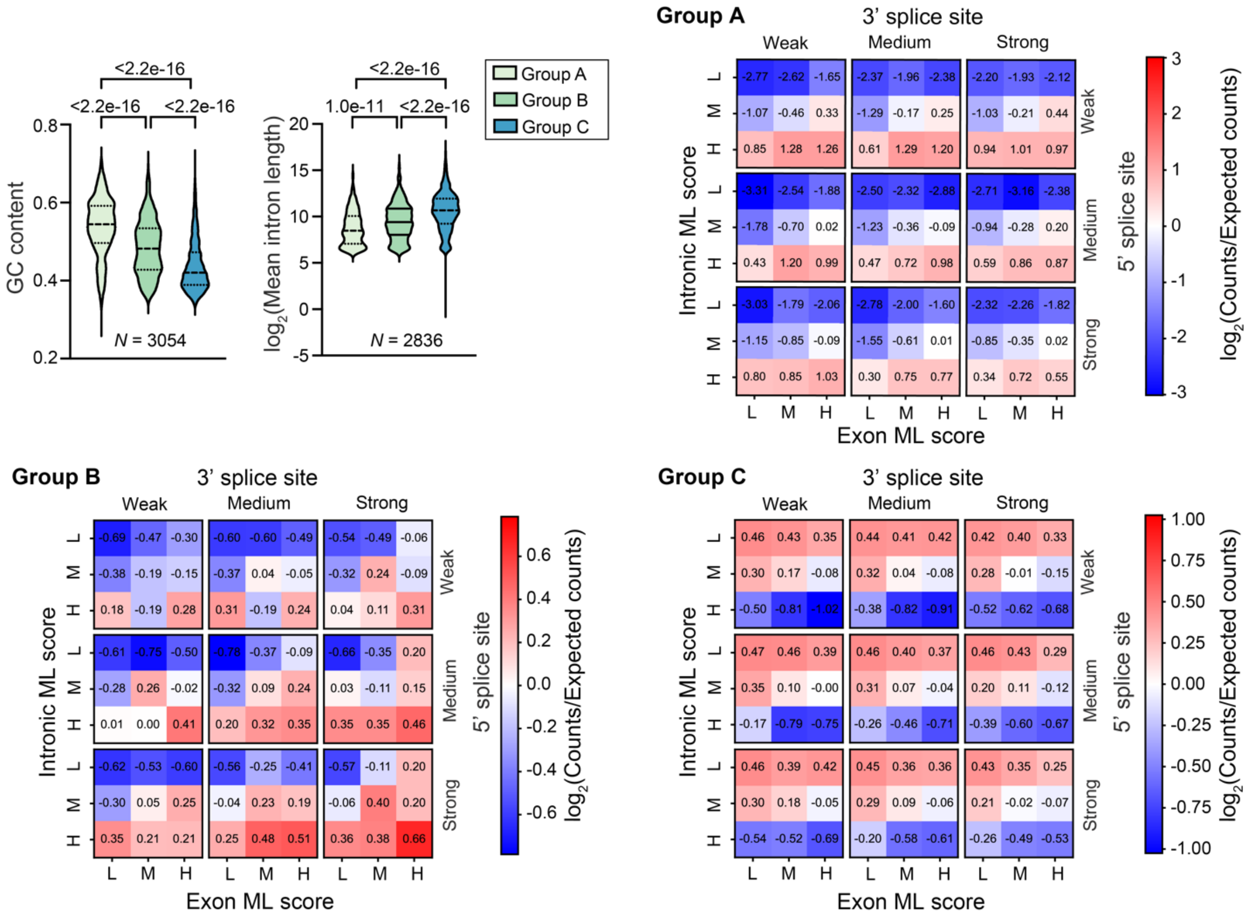

Figure S12: Comparison of GC content, intron length, and splicing-related features in Group A, B, and C transcripts. GC content and intron length of Group A, B and C genes are shown in violin plots. *P*-values are calculated using unpaired t-test. Splicing-related features represented as a combination of ML score with splice site strength for Group A, B and C genes are shown in heatmaps. The color bar indicates the $\log_2$ fold change of counts of each splicing-related combination compared to expected counts. Blue: a combination with lower-than-expected count (depleted); red: a combination with higher-than-expected count (enriched).

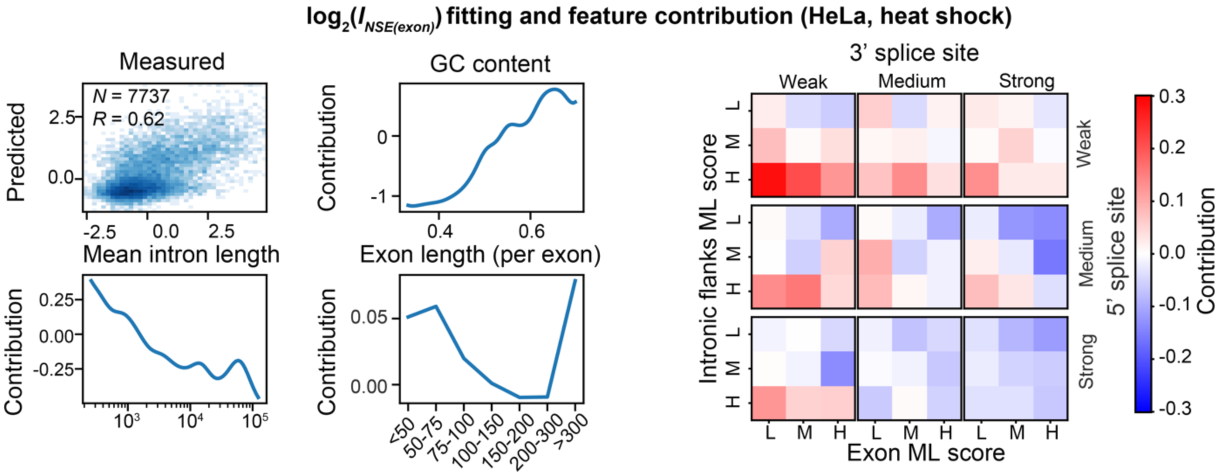

Figure S13: Regression analysis on transcript speckle enrichment under heat shock. The exon-centric regression analysis on $I_{NSE(exon)}$ values in HeLa cells upon heat shock. "*N*" reports the number of genes in the regression analysis, and "*R*" reports Pearson's correlation coefficient.

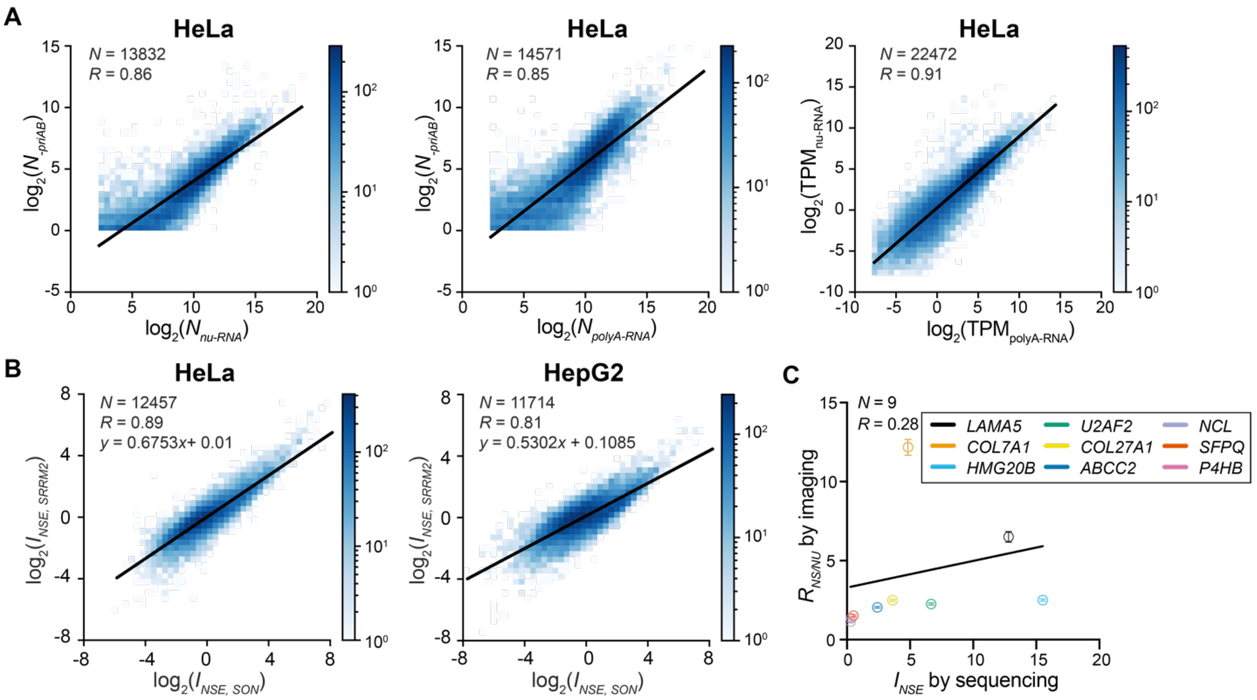

Figure S14. I_{NSE} calculation using normalization to nuclear RNA-seq. (A) 2D histogram showing the correlation between mapped reads to each gene from ARTR-seq without primary antibody (N_{-priAB}) and rRNA-depleted nuclear-RNA seq ($N_{nu\text{-}RNA}$) (left) or polyA RNA-seq ($N_{\text{polyA-RNA}}$) (middle), and the correlation between TPM (in $\log_2$ scale) from nuclear RNA-seq ($TPM_{\text{nu-RNA}}$) and TPM (in $\log_2$ scale) from polyA RNA-seq ($TPM_{\text{polyA-RNA}}$) (right). (B) 2D histogram showing the correlation between I_{NSE} determined through targeting SON and SRRM2 in HeLa and HepG2 cells. I_{NSE} was calculated by differential analysis between a background corrected N_{SON} (or N_{SRRM2}) and $N_{nu\text{-}RNA}$. Genes with $lfcSE<1$ from DESeq analysis of ARTR-seq are included. Linear function ($y = ax+b$) is used to fit $I_{NSE,\ SRRM2}$ to $I_{NSE,SON}$ and labeled in each 2D histogram. (C) Correlation between I_{NSE} and $R_{NS/NU}$ values determined by RNA FISH imaging as in Figures 1E and S1C. Since $N_{nu\text{-}RNA}$ reflects the nuclear RNA abundance, I_{NSE} calculated using normalization to $N_{nu\text{-}RNA}$ reports the ratio of RNA concentration in nuclear speckles to the RNA concentration in nucleoplasm. $R_{NS/NU}$ value is defined by the ratio of RNA intensity in speckles to the RNA concentration in the nucleoplasm, equivalent to I_{NSE} determined using this normalization method. "N" reports the total number of genes included in each plot, and "R" reports Pearson's correlation coefficient.

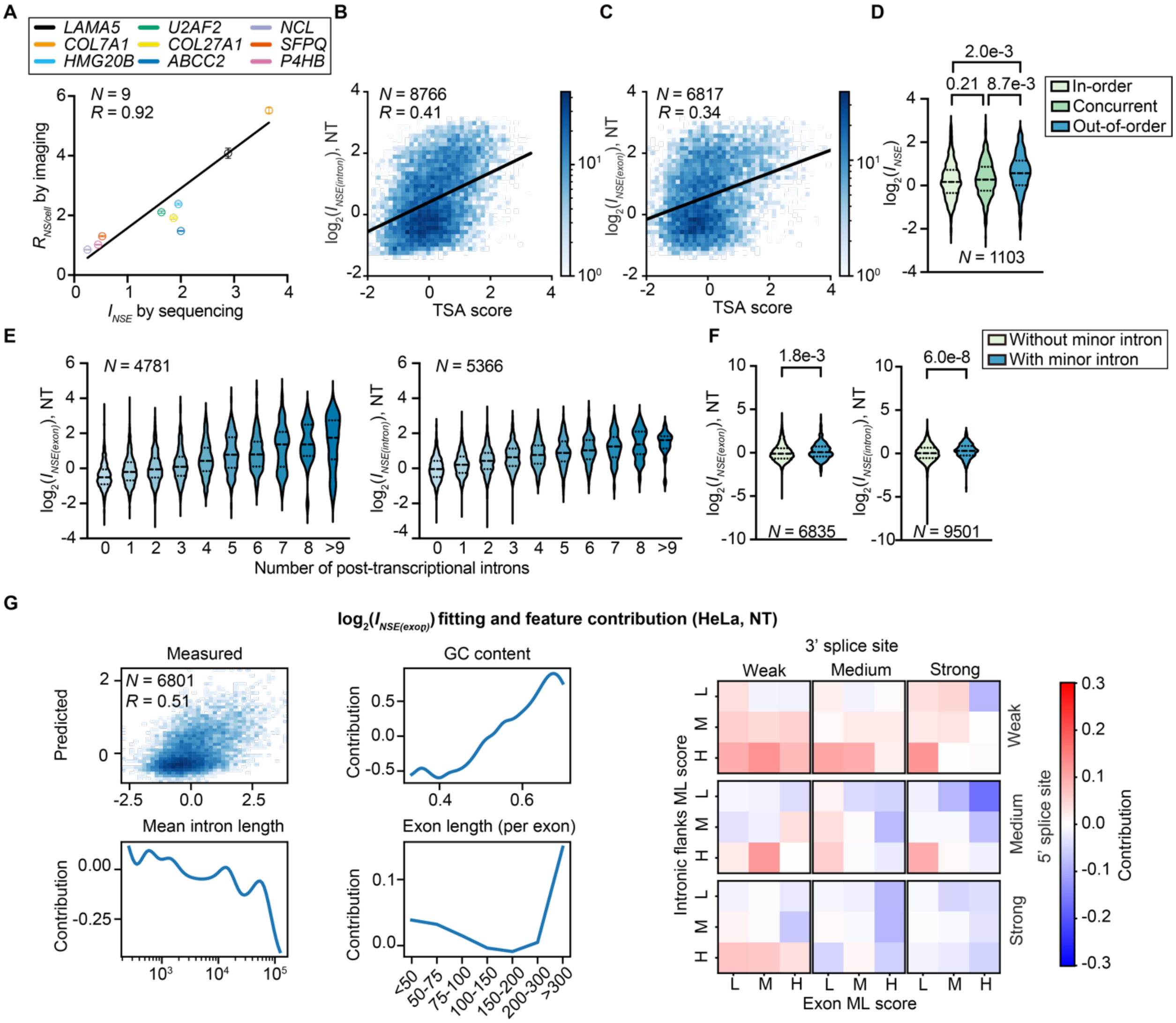

Figure S15. Characterization of nuclear speckle-enriched transcriptome using ARTR-seq with SRRM2 antibody. (A) Correlation between speckle partition coefficient ($R_{NS/cell}$) measured by RNA FISH imaging and I_{NSE} values determined by ARTR-seq using SRRM2 antibody. $R_{NS/cell}$ is calculated as described in Figure 1F. (B-C) 2D histogram showing the correlation between $I_{NSE(intron)}$ (B) or $I_{NSE(exon)}$ (C) in HeLa cells and TSA score from TSA-seq in K562 cells. Genes with higher TSA scores are closer to speckles. (D) Violin plot comparing I_{NSE} values of in-order excised introns, concurrently excised introns, and out-of-order excised introns, as classified by CoLa-seq. (E) Violin plot comparing $I_{NSE(exon)}$ or $I_{NSE(intron)}$ values of transcripts containing different numbers of post-transcriptionally excised introns, as characterized by nanopore RNA-seq. (F) Violin plot comparing $I_{NSE(exon)}$ or $I_{NSE(intron)}$ values of transcripts containing minor splice sites and those without. (G) The exon-centric regression model reveals contributions from GC content, mean intron length, individual exon length, and a combination of splice site strength, exonic ML score and flanking intronic ML score to the transcript speckle enrichment $I_{NSE(exon)}$ values under NT in HeLa cells. "*N*" reports the number of genes included in the analysis in (A)-(C), (E) and (G), and number of introns in (D) and (F). "*R*" reports Pearson's correlation coefficient. *P*-values calculated with unpaired t-tests are reported above each violin plot in (D) and (F).

Supplementary tables

Table S1. Group A, B and C gene list.
Table S2. List of oligos used in this study.
Table S3. DEseq2 file in HeLa cells.
Table S4. DEseq2 file in HepG2 cells.
Table S5. Mapped reads analysis.
Table S6. IRFinder analysis.

REFERENCES AND NOTES

1. T. Hirose, K. Ninomiya, S. Nakagawa, T. Yamazaki, A guide to membraneless organelles and their various roles in gene regulation. *Nat. Rev. Mol. Cell Biol.* **24**, 288–304 (2023).

2. L. Galganski, M. O. Urbanek, W. J. Krzyzosiak, Nuclear speckles: Molecular organization, biological function and role in disease. *Nucleic Acids Res.* **45**, 10350–10368 (2017).

3..A Ilık, T. Aktaş, Nuclear speckles: Dynamic hubs of gene expression regulation. *FEBS J.* **289**, 7234–7245 (2022).

4..A Ilik, M. Malszycki, A. K. Lübke, C. Schade, D. Meierhofer, T. Aktaş, SON and SRRM2 are essential for nuclear speckle formation. *eLife* **9**, e60579 (2020).

5. A. R. Barutcu, M. Wu, U. Braunschweig, B. J. A. Dyakov, Z. Luo, K. M. Turner, T. Durbic, Z.-Y. Lin, R. J. Weatheritt, P. G. Maass, A.-C. Gingras, B. J. Blencowe, Systematic mapping of nuclear domain-associated transcripts reveals speckles and lamina as hubs of functionally distinct retained introns. *Mol. Cell* **82**, 1035–1052.e9 (2022).

6. E. Lester, F. K. Ooi, N. Bakkar, J. Ayers, A. L. Woerman, J. Wheeler, R. Bowser, G. A. Carlson, S. B. Prusiner, R. Parker, Tau aggregates are RNA-protein assemblies that mislocalize multiple nuclear speckle components. *Neuron* **109**, 1675–1691.e9 (2021).

7. M. O. Urbanek, M. Jazurek, P. M. Switonski, G. Figura, W. J. Krzyzosiak, Nuclear speckles are detention centers for transcripts containing expanded CAG repeats. *Biochim. Biophys. Acta* **1862**, 1513–1520 (2016).

8. A. Mor, A. White, K. Zhang, M. Thompson, M. Esparza, R. Muñoz-Moreno, K. Koide, K. W. Lynch, A. García-Sastre, B. M. A. Fontoura, Influenza virus mRNA trafficking through host nuclear speckles. *Nat. Microbiol.* **1**, 1–13 (2016).

9. Y. Chen, A. S. Belmont, Genome organization around nuclear speckles. *Curr. Opin. Genet. Dev.* **55**, 91–99 (2019).

10. L. L. Hall, K. P. Smith, M. Byron, J. B. Lawrence, Molecular anatomy of a speckle. *Anat. Rec. A Discov. Mol. Cell. Evol. Biol.* **288**, 664–675 (2006).

11. F. Ding, M. B. Elowitz, Constitutive splicing and economies of scale in gene expression. *Nat. Struct. Mol. Biol.* **26**, 424–432 (2019).

12. W. Chen, Z. Yan, S. Li, N. Huang, X. Huang, J. Zhang, S. Zhong, RNAs as proximity-labeling media for identifying nuclear speckle positions relative to the genome. *iScience* **4**, 204–215 (2018).

13. Y. Chen, Y. Zhang, Y. Wang, L. Zhang, E. K. Brinkman, S. A. Adam, R. Goldman, B. van Steensel, J. Ma, A. S. Belmont, Mapping 3D genome organization relative to nuclear compartments using TSA-Seq as a cytological ruler. *J. Cell Biol.* **217**, 4025–4048 (2018).

14. S. A. Quinodoz, N. Ollikainen, B. Tabak, A. Palla, J. M. Schmidt, E. Detmar, M. M. Lai, A. A. Shishkin, P. Bhat, Y. Takei, V. Trinh, E. Aznauryan, P. Russell, C. Cheng, M. Jovanovic, A. Chow, L. Cai, P. McDonel, M. Garber, M. Guttman, Higher-order inter-chromosomal hubs shape 3D genome organization in the nucleus. *Cell* **174**, 744–757.e24 (2018).

15. J. M. Brown, J. Leach, J. E. Reittie, A. Atzberger, J. Lee-Prudhoe, W. G. Wood, D. R. Higgs, F. J. Iborra, V. J. Buckle, Coregulated human globin genes are frequently in spatial proximity when active. *J. Cell Biol.* **172**, 177–187 (2006).

16. N. Khanna, Y. Hu, A. S. Belmont, HSP70 transgene directed motion to nuclear speckles facilitates heat shock activation. *Curr. Biol.* **24**, 1138–1144 (2014).

17. K. A. Alexander, A. Coté, S. C. Nguyen, L. Zhang, O. Gholamalamdari, P. Agudelo-Garcia, E. Lin-Shiao, K. M. A. Tanim, J. Lim, N. Biddle, M. C. Dunagin, C. R. Good, M. R. Mendoza, S. C. Little, A. Belmont, E. F. Joyce, A. Raj, S. L. Berger, p53 mediates target gene association with nuclear speckles for amplified RNA expression. *Mol. Cell* **81**, 1666–1681.e6 (2021).

18. M. Hluchý, P. Gajdušková, I. R. de los Mozos, M. Rájecký, M. Kluge, B.-T. Berger, Z. Slabá, D. Potěšil, E. Weiß, J. Ule, Z. Zdráhal, S. Knapp, K. Paruch, C. C. Friedel, D. Blazek, CDK11 regulates pre-mRNA splicing by phosphorylation of SF3B1. *Nature* **609**, 829–834 (2022).

19. Y. Shi, B. Reddy, J. L. Manley, PP1/PP2A phosphatases are required for the second step of pre-mRNA splicing and target specific snRNP proteins. *Mol. Cell* **23**, 819–829 (2006).

20. C. Wang, K. Chua, W. Seghezzi, E. Lees, O. Gozani, R. Reed, Phosphorylation of spliceosomal protein SAP 155 coupled with splicing catalysis. *Genes Dev.* **12**, 1409–1414 (1998).

21. C. Girard, C. L. Will, J. Peng, E. M. Makarov, B. Kastner, I. Lemm, H. Urlaub, K. Hartmuth, R. Lührmann, Post-transcriptional spliceosomes are retained in nuclear speckles until splicing completion. *Nat. Commun.* **3**, 994 (2012).

22. D. Kaida, H. Motoyoshi, E. Tashiro, T. Nojima, M. Hagiwara, K. Ishigami, H. Watanabe, T. Kitahara, T. Yoshida, H. Nakajima, T. Tani, S. Horinouchi, M. Yoshida, Spliceostatin A targets SF3b and inhibits both splicing and nuclear retention of pre-mRNA. *Nat. Chem. Biol.* **3**, 576–583 (2007).

23. P. Bhat, A. Chow, B. Emert, O. Ettlin, S. A. Quinodoz, M. Strehle, Y. Takei, A. Burr, I. N. Goronzy, A. W. Chen, W. Huang, J. L. M. Ferrer, E. Soehalim, S.-T. Goh, T. Chari, D. K. Sullivan, M. R. Blanco, M. Guttman, Genome organization around nuclear speckles drives mRNA splicing efficiency. *Nature* **629**, 1165–1173 (2024).

24. H.-M. Sung, J. Schott, P. Boss, J. A. Lehmann, M. R. Hardt, D. Lindner, J. Messens, I. Bogeski, U. Ohler, G. Stoecklin, Stress-induced nuclear speckle reorganization is linked to activation of immediate early gene splicing. *J. Cell Biol.* **222**, e202111151 (2023).

25. S. Xu, S.-K. Lai, D. Y. Sim, W. S. L. Ang, H. Y. Li, X. Roca, SRRM2 organizes splicing condensates to regulate alternative splicing. *Nucleic Acids Res.* **50**, 8599–8614 (2022).

26. Y. Xiao, Y.-M. Chen, Z. Zou, C. Ye, X. Dou, J. Wu, C. Liu, S. Liu, H. Yan, P. Wang, T.-B. Zeng, Q. Liu, J. Fei, W. Tang, C. He, Profiling of RNA-binding protein binding sites by in situ reverse transcription-based sequencing. *Nat. Methods* **21**, 247–258 (2024).

27. Y. Zou, B. Tu, L. Yu, Y. Zheng, Y. Lin, W. Luo, Y. Yang, Q. Fang, C. Wang, Peptide conformation and oligomerization characteristics of surface-mediated assemblies revealed by molecular dynamics simulations and scanning tunneling microscopy. *RSC Adv.* **9**, 41345–41350 (2019).

28. J. N. Hutchinson, A. W. Ensminger, C. M. Clemson, C. R. Lynch, J. B. Lawrence, A. Chess, A screen for nuclear transcripts identifies two linked noncoding RNAs associated with SC35 splicing domains. *BMC Genomics* **8**, 39 (2007).

29. J. Dopie, M. J. Sweredoski, A. Moradian, A. S. Belmont, Tyramide signal amplification mass spectrometry (TSA-MS) ratio identifies nuclear speckle proteins. *J. Cell Biol.* **219**, e201910207 (2020).

30. S. Paul, M. A. Arias, L. Wen, S. E. Liao, J. Zhang, X. Wang, O. Regev, J. Fei, RNA molecules display distinctive organization at nuclear speckles. *iScience* **27**, 109603 (2024).

31. T. Carvalho, S. Martins, J. Rino, S. Marinho, M. Carmo-Fonseca, Pharmacological inhibition of the spliceosome subunit SF3b triggers EJC-independent NMD. *J. Cell Sci.* **130**, 1519–1531 (2017).

32. K. B. Chapman, J. D. Boeke, Isolation and characterization of the gene encoding yeast debranching enzyme. *Cell* **65**, 483–492 (1991).

33. L. Herzel, D. S. M. Ottoz, T. Alpert, K. M. Neugebauer, Splicing and transcription touch base: Co-transcriptional spliceosome assembly and function. *Nat. Rev. Mol. Cell Biol.* **18**, 637–650 (2017).

34. M. Brugiolo, L. Herzel, K. M. Neugebauer, Counting on co-transcriptional splicing. *F1000Prime Rep.* **5**, 9 (2013).

35. R. Reed, E. Hurt, A conserved mRNA export machinery coupled to pre-mRNA splicing. *Cell* **108**, 523–531 (2002).

36. P. Valencia, A. P. Dias, R. Reed, Splicing promotes rapid and efficient mRNA export in mammalian cells. *Proc. Natl. Acad. Sci. U.S.A.* **105**, 3386–3391 (2008).

37. Y. Wang, W. T. Cottle, H. Wang, X. A. Feng, J. Mallon, M. Gavrilov, S. Bailey, T. Ha, Genome oligopaint via local denaturation fluorescence in situ hybridization. *Mol. Cell* **81**, 1566–1577.e8 (2021).

38. R. Andersson, P. Refsing Andersen, E. Valen, L. J. Core, J. Bornholdt, M. Boyd, T. Heick Jensen, A. Sandelin, Nuclear stability and transcriptional directionality separate functionally distinct RNA species. *Nat. Commun.* **5**, 5336 (2014).

39. Y. Zeng, B. J. Fair, H. Zeng, A. Krishnamohan, Y. Hou, J. M. Hall, A. J. Ruthenburg, Y. I. Li, J. P. Staley, Profiling lariat intermediates reveals genetic determinants of early and late co-transcriptional splicing. *Mol. Cell* **82**, 4681–4699.e8 (2022).

40. K. Choquet, A. R. Baxter-Koenigs, S.-L. Dülk, B. M. Smalec, S. Rouskin, L. S. Churchman, Pre-mRNA splicing order is predetermined and maintains splicing fidelity across multi-intronic transcripts. *Nat. Struct. Mol. Biol.* **30**, 1064–1076 (2023).

41. A. A. Patel, M. McCarthy, J. A. Steitz, The splicing of U12-type introns can be a rate-limiting step in gene expression. *EMBO J.* **21**, 3804–3815 (2002).

42. A. M. Olthof, K. C. Hyatt, R. N. Kanadia, Minor intron splicing revisited: Identification of new minor intron-containing genes and tissue-dependent retention and alternative splicing of minor introns. *BMC Genomics* **20**, 686 (2019).

43. H. Hochberg-Laufer, N. Neufeld, Y. Brody, S. Nadav-Eliyahu, R. Ben-Yishay, Y. Shav-Tal, Availability of splicing factors in the nucleoplasm can regulate the release of mRNA from the gene after transcription. *PLOS Genet.* **15**, e1008459 (2019).

44. G. Yeo, C. B. Burge, Maximum entropy modeling of short sequence motifs with applications to RNA splicing signals. *J. Comput. Biol.* **11**, 377–394 (2004).

45. S. E. Liao, M. Sudarshan, O. Regev, Deciphering RNA splicing logic with interpretable machine learning. *Proc. Natl. Acad. Sci. U.S.A.* **120**, e2221165120 (2023).

46. M. A. Arias, A. Lubkin, L. A. Chasin, Splicing of designer exons informs a biophysical model for exon definition. *RNA* **21**, 213–229 (2015).

47. Z. Dominski, R. Kole, Selection of splice sites in pre-mRNAs with short internal exons. *Mol. Cell. Biol.* **11**, 6075–6083 (1991).

48. U. Braunschweig, N. L. Barbosa-Morais, Q. Pan, E. N. Nachman, B. Alipanahi, T. Gonatopoulos-Pournatzis, B. Frey, M. Irimia, B. J. Blencowe, Widespread intron retention in mammals functionally tunes transcriptomes. *Genome Res.* **24**, 1774–1786 (2014).

49. R. Middleton, D. Gao, A. Thomas, B. Singh, A. Au, J. J.-L. Wong, A. Bomane, B. Cosson, E. Eyras, J. E. J. Rasko, W. Ritchie, IRFinder: Assessing the impact of intron retention on mammalian gene expression. *Genome Biol.* **18**, 51 (2017).

50. G. Monteuuis, J. J. L. Wong, C. G. Bailey, U. Schmitz, J. E. J. Rasko, The changing paradigm of intron retention: Regulation, ramifications and recipes. *Nucleic Acids Res.* **47**, 11497–11513 (2019).

51. D. S. W. Protter, R. Parker, Principles and properties of stress granules. *Trends Cell Biol.* **26**, 668–679 (2016).

52. C. L. Riggs, N. Kedersha, P. Ivanov, P. Anderson, Mammalian stress granules and P bodies at a glance. *J. Cell Sci.* **133**, jcs242487 (2020).

53. P. L. Boutz, A. Bhutkar, P. A. Sharp, Detained introns are a novel, widespread class of post-transcriptionally spliced introns. *Genes Dev.* **29**, 63–80 (2015).

54. J. T. Morgan, G. R. Fink, D. P. Bartel, Excised linear introns regulate growth in yeast. *Nature* **565**, 606–611 (2019).

55. J. Parenteau, L. Maignon, M. Berthoumieux, M. Catala, V. Gagnon, S. Abou Elela, Introns are mediators of cell response to starvation. *Nature* **565**, 612–617 (2019).

56. R. Shalgi, J. A. Hurt, S. Lindquist, C. B. Burge, Widespread inhibition of posttranscriptional splicing shapes the cellular transcriptome following heat shock. *Cell Rep.* **7**, 1362–1370 (2014).

57. H. H. Kampinga, J. Hageman, M. J. Vos, H. Kubota, R. M. Tanguay, E. A. Bruford, M. E. Cheetham, B. Chen, L. E. Hightower, Guidelines for the nomenclature of the human heat shock proteins. *Cell Stress Chaperones* **14**, 105–111 (2009).

58. D. B. Mahat, H. H. Salamanca, F. M. Duarte, C. G. Danko, J. T. Lis, Mammalian heat shock response and mechanisms underlying its genome-wide transcriptional regulation. *Mol. Cell* **62**, 63–78 (2016).

59. L. Tammer, O. Hameiri, I. Keydar, V. R. Roy, A. Ashkenazy-Titelman, N. Custódio, I. Sason, R. Shayevitch, V. Rodríguez-Vaello, J. Rino, G. L. Maor, Y. Leader, D. Khair, E. L. Aiden, R. Elkon, M. Irimia, R. Sharan, Y. Shav-Tal, M. Carmo-Fonseca, G. Ast, Gene architecture directs splicing outcome in separate nuclear spatial regions. *Mol. Cell* **82**, 1021–1034.e8 (2022).

60. A. Jain, R. D. Vale, RNA phase transitions in repeat expansion disorders. *Nature* **546**, 243–247 (2017).

61. S. Wang, Y. Xu, RNA structure promotes liquid-to-solid phase transition of short RNAs in neuronal dysfunction. *Commun. Biol.* **7**, 1–14 (2024).

62. X.-D. Fu, M. Ares, Context-dependent control of alternative splicing by RNA-binding proteins. *Nat. Rev. Genet.* **15**, 689–701 (2014).

63. C. Johnson, D. Primorac, M. McKinstry, J. McNeil, D. Rowe, J. B. Lawrence, Tracking Col1a1 RNA in osteogenesis imperfecta: Splice-defective transcripts initiate transport from the gene but are retained within the Sc35 domain. *J. Cell Biol.* **150**, 417–432 (2000).

64. S. Irgen-Gioro, S. Yoshida, V. Walling, S. Chong, Fixation can change the appearance of phase separation in living cells. *eLife* **11**, e79903 (2022).

65. L. Vigevani, A. Gohr, T. Webb, M. Irimia, J. Valcárcel, Molecular basis of differential 3′ splice site sensitivity to anti-tumor drugs targeting U2 snRNP. *Nat. Commun.* **8**, 2100 (2017).

66. Y. Kotake, K. Sagane, T. Owa, Y. Mimori-Kiyosue, H. Shimizu, M. Uesugi, Y. Ishihama, M. Iwata, Y. Mizui, Splicing factor SF3b as a target of the antitumor natural product pladienolide. *Nat. Chem. Biol.* **3**, 570–575 (2007).

67. J. Schindelin, I. Arganda-Carreras, E. Frise, V. Kaynig, M. Longair, T. Pietzsch, S. Preibisch, C. Rueden, S. Saalfeld, B. Schmid, J.-Y. Tinevez, D. J. White, V. Hartenstein, K. Eliceiri, P. Tomancak, A. Cardona, Fiji: An open-source platform for biological-image analysis. *Nat. Methods* **9**, 676–682 (2012).

68. M. Martin, Cutadapt removes adapter sequences from high-throughput sequencing reads. *EMBnet. J.* **17**, 10–12 (2011).

69. A. Dobin, C. A. Davis, F. Schlesinger, J. Drenkow, C. Zaleski, S. Jha, P. Batut, M. Chaisson, T. R. Gingeras, STAR: Ultrafast universal RNA-seq aligner. *Bioinformatics* **29**, 15–21 (2013).

70. Y. Liao, G. K. Smyth, W. Shi, FeatureCounts: An efficient general purpose program for assigning sequence reads to genomic features. *Bioinformatics* **30**, 923–930 (2014).

71. B. Li, C. N. Dewey, RSEM: Accurate transcript quantification from RNA-Seq data with or without a reference genome. *BMC Bioinform.* **12**, 323 (2011).

72. M. I. Love, W. Huber, S. Anders, Moderated estimation of fold change and dispersion for RNA-seq data with DESeq2. *Genome Biol.* **15**, 550 (2014).

73. L. Kolberg, U. Raudvere, I. Kuzmin, P. Adler, J. Vilo, H. Peterson, g:Profiler—Interoperable web service for functional enrichment analysis and gene identifier mapping. *Nucleic Acids Res.* **51**, W207–W212 (2023).

74. D. Servén, C. Brummitt, pyGAM: Generalized Additive Models in Python, version v0.4.1 (2018); https://doi.org/10.5281/zenodo.1208724.

75. I. Gaspar, F. Wippich, A. Ephrussi, Enzymatic production of single-molecule FISH and RNA capture probes. *RNA* **23**, 1582–1591 (2017).

76. J. Rouillard, M. Zuker, E. Gulari, OligoArray 2.0: Design of oligonucleotide probes for DNA microarrays using a thermodynamic approach. *Nucleic Acids Res.* **31**, 3057–3062 (2003).

77. D. D. Kocak, E. A. Josephs, V. Bhandarkar, S. S. Adkar, J. B. Kwon, C. A. Gersbach, Increasing the specificity of CRISPR systems with engineered RNA secondary structures. *Nat. Biotechnol.* **37**, 657–666 (2019).

78. C. Stringer, T. Wang, M. Michaelos, M. Pachitariu, Cellpose: A generalist algorithm for cellular segmentation. *Nat. Methods* **18**, 100–106 (2021).

79. M. Pachitariu, C. Stringer, Cellpose 2.0: How to train your own model. *Nat. Methods* **19**, 1634–1641 (2022).

80. A. C. Francis, M. Marin, P. K. Singh, V. Achuthan, M. J. Prellberg, K. Palermino-Rowland, S. Lan, P. R. Tedbury, S. G. Sarafianos, A. N. Engelman, G. B. Melikyan, HIV-1 replication complexes accumulate in nuclear speckles and integrate into speckle-associated genomic domains. *Nat. Commun.* **11**, 3505 (2020).

81. U. Schmidt, M. Weigert, C. Broaddus, G. Myers, "Cell detection with star-convex polygons" in *Medical Image Computing and Computer Assisted Intervention*, A. F. Frangi, J. A. Schnabel, C. Davatzikos, C. Alberola-López, G. Fichtinger, Eds. (Springer, 2018), pp. 265–273.

82. U. Raudvere, L. Kolberg, I. Kuzmin, T. Arak, P. Adler, H. Peterson, J. Vilo, g:Profiler: A web server for functional enrichment analysis and conversions of gene lists. *Nucleic Acids Res.* **47**, W191–W198 (2019).
